# Synthesis and Spectral Properties of Novel Singapore Green Analogues for Protease Detection

**DOI:** 10.1038/s41598-019-57124-0

**Published:** 2020-01-14

**Authors:** Luke Bywaters, Adam Le Gresley

**Affiliations:** School of Life Sciences, Pharmacy and Chemistry, SEC Faculty, Kingston University, Kingston-upon-Thames, KT1 2EE UK

**Keywords:** Chemistry, Organic chemistry, Photochemistry, Chemical synthesis

## Abstract

Herein we describe the synthesis, characterisation and determination of fluorescence and photophysical properties of various novel analogues of the orphan fluorophore class Singapore Green. We equate the fluorescence properties of these novel fluorophores to their molecular structure and address the mechanisms through which their fluorescence is quenched and the effect this has on their quantum yields of fluorescence. Fluorescence quenching *via* acylation was also achieved, thereby providing conceptual proof of their utility as cores for future fluorescent probes. Additionally, we have produced and examined a number of unexpected acyl intermediates of variable photolytic stability. Furthermore, we have obtained proof of concept that the use of Singapore Greens for protease probe generation is feasible *via* demonstration of proteolytic cleavage of one of the acylated analogues.

## Introduction

Many clinically significant events involve changes in concentrations of proteases, which can be a useful handle in the detection of problem pathogens such as ESBL resistant bacteria and carbapenemase producing bacteria^1,2^. Conventional detection strategies e.g. cell culture & PCR require skilled technicians and relatively long time periods (24–72 hrs in clinic) to return their respective results. Indeed, in the UK, Polymerase Chain Reaction (PCR) is not used in hospitals for routine screening owing to the prohibitive cost and protracted bacteriological culture is the default procedure for the detection of problem pathogens in healthcare and other environments. This point has been highlighted in the World Health Organisation’s recent report stating that “Governments and partners need to work closely with industry to encourage greater investment in research and development of new diagnostics that can improve decision making”^3^.

Fluorescent probes possessing both a high quantum yield of fluorescence (QY) and large extinction coefficient (ε) have garnered a lot of attention in the past decades and are currently enjoying a resurgence owing to their relative simplicity and their potential application at the point of decision-making^4–8^. Properly derivatised with a target peptide, these probes possess the ability to switch from very low fluorescence to very high fluorescence upon exposure, therefore leading to high contrast and hence sensitivity. Their increasing importance is not only due to their high sensitivity, but also the rapidity of generation of a positive result^9,10^.

Fluorescent probes commonly work *via* attachment of a particular biomimetic to a fluorophore, the biomimetic is often a peptide substrate of a protease enzyme expressed by the pathogen of interest^9^. When the peptide is attached, the fluorescence of the probe is quenched, usually *via* the ability to shed electronic excitation energy through collisional, thermal and other non-radiative means. However, upon cleavage of the peptide sequence the active fluorophore is released and fluorescence is observed, signalling a positive result. A large proportion of these probes are based on a relatively small number of fluorophores, commonly rhodamines and fluoresceins. Although several new classes of fluorophores with various properties have been developed, there remains much scope for improvement in order to expand the existing fluorescent detection toolkit^11,12^.

A new class of fluorophore known as Singapore Green was produced by Li *et al*.^13^ It enjoyed similar properties to that of both rhodamines and fluoresceins, while possessing both an amino group and a hydroxy group, either of which could be functionalised for probe generation. In theory it could provide an effective substitute for either of these fluorophores. The advantage of such a substitution however, is that, due to its non-symmetrical nature, only single cleavage event would be required to produce full fluorescence. Furthermore, simplification of probe production would be enjoyed upon its utilisation, as only a single biomimetic arm is required to be attached for substrate targeting. This is in addition to it lacking the often troublesome upper carboxyl group. As we have previously shown, both bis acylation of rhodamine 110’s amino groups and the presence of the upper carboxyl group can complicate probe production^15^. To facilitate biological research requirements, we herein employ a selection of Grignard reagents to add to and enrich the understanding of this important class of fluorophore.

## Results and Discussion

**Scheme 1:**
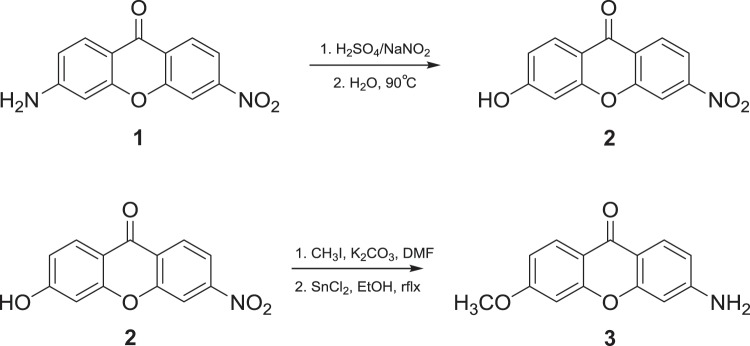
Synthetic conditions employed for production of xanthone precursors, as per Ahn *et al*.^14^.

**Scheme 2:**
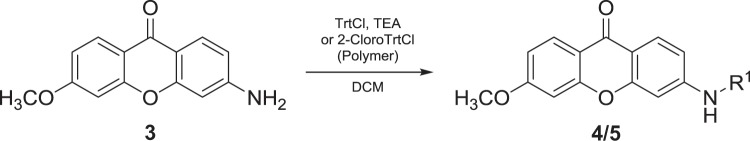
Anilino protection, where R^1^ = Trt = (**4**) or R^1^ = polystyrene bound 2-chloroTrt = (**5**).

**Scheme 3:**
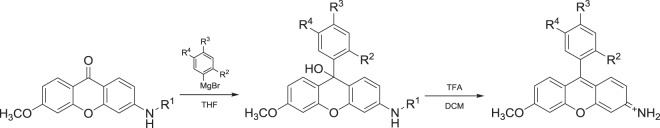
Addition of Grignard reagent to the trityl protected xanthone precursor (**4**), to form tertiary alcohol (**6**) or (**7**). Addition to precursor (**5**) for tertiary alcohols (**8**) – (**14**), where R^1^/R^2^/R^3^/R^4^ are shown in Table 1. Deprotection to form Singapore Greens (**15) – (23)** where R2/R3/R4 are shown in Table 2.

Work was initiated on the synthesis of analogues of Singapore Green (SG) starting from the precursor xanthones using proven methodology as per Ahn *et al*. and further by Li *et al*. (Schemes 1–3)^11,14^. Details of all synthetic procedures are included in the synthetic methods section.

**Table 1:** Substituents of tertiary alcohols (**6**) – (**14**) generated following Grignard addition.

Compound	R^1^	R^2^	R^3^	R^4^
6	Trt	CH_3_	H	H
7	Trt	CF_3_	H	H
8	(poly)-2-ChloroTrt	CH_3_	H	CH_3_
9	(poly)-2-ChloroTrt	OCH_3_	H	H
10	(poly)-2-ChloroTrt	OCH_3_	H	CH_3_
11	(poly)-2-ChloroTrt	CH_3_	CH_3_	H
12	(poly)-2-ChloroTrt	OCH_3_	OCH_3_	H
13	(poly)-2-ChloroTrt	C_4_H_4_	H	H
14	(poly)-2-ChloroTrt	CF_3_	H	H

**Table 2:** Substituents of Singapore Greens (**15**) – (**23**) generated following deprotection.

Compound	R^2^	R^3^	R^4^
15	CH_3_	H	H
16	CF_3_	H	H
17	CH_3_	H	CH_3_
18	OCH_3_	H	H
19	OCH_3_	H	CH_3_
20	CH_3_	CH_3_	H
21	OCH_3_	OCH_3_	H
22	C_4_H_4_	H	H
23	CF_3_	H	H.

Following production of the parent xanthone (**3**) the reactive anilino NH_2_ group required base stable protection. The trityl group was selected for this purpose while undergoing Grignard addition. Following formation of tertiary alcohol (**6**), the trityl group was removed using standard methodology, namely 1:1 TFA:DCM.

Purification was then attempted *via* silica chromatography using the same procedure as stated in the literature^13^. In our hands however, purification of this compound was not possible as a result of deprotection occurring on the silica *in situ*. It is noteworthy that although tertiary alcohol (**6**) was stated to have been isolated in the original report, no spectroscopic evidence was provided^13^. In light of our observations, trityl deprotection of the tertiary alcohol was undertaken immediately following Grignard addition and workup, reducing the number of synthetic steps. Silica chromatography followed to yield the target product Singapore Green^13^ in a modest yield of ~8%.

Having demonstrated the viability of the shortened synthetic procedure and reproduced the orphan Singapore Green fluorophore (**15**) and novel analogue (**16**), further synthetic simplification was sought so as to aid the production of further novel analogues. In light of the ready availability of trityl functionalised polystyrene resins, along with the purported increased ease of handling of such bound compounds, a solid supported strategy was adopted^14^.

**Figure 1:**
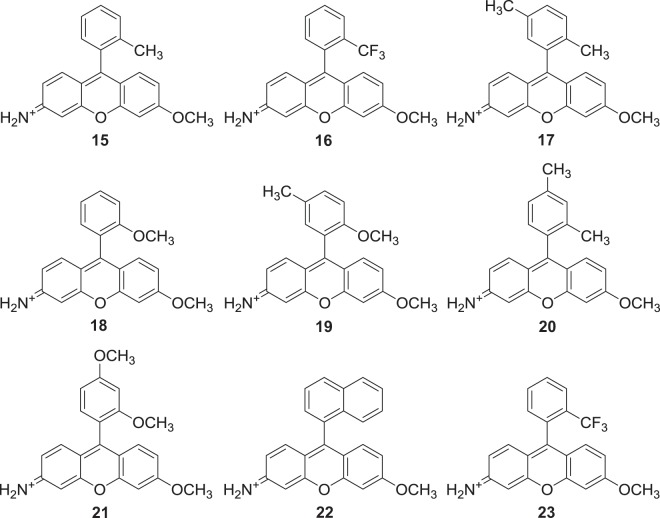
The structures of Singapore Greens (**15**) – (**23**). N.B. Counter ion for (**15**) and (**17**) – (**23**) = TFA^−^, (**16**) = Cl^−^.

Using solid supported xanthone (**5**) a library of novel Singapore Green analogues (**17**) - (**23**) were generated in modest yield and isolated using our modified procedure as before. Having successfully prepared the original Singapore Green (**15**) and a collection of novel analogues (**16**) - (**23**) (Fig. 1), investigation into the effects that substitution of the upper phenyl ring had on their optical properties commenced. In order to achieve this the relative fluorescence quantum yields (QY), extinction coefficients (ε) and excitation and emission maxima (λ_ex_/λ_em_) were determined Figures.

It was postulated in the seminal paper by Urano *et al*. that, contrary to previous research, the upper carboxylic acid group of fluorescein was not essential for strong fluorescence^15^. Indeed, it only acted as a steric barrier to rotation, and this was demonstrated by substitution for other sterically hindering groups such as CH_3_. The result of this substitution was to produce a new class of highly fluorescent molecule, the so-called Tokyo Greens. These possessed near identical fluorescence and optical properties to that of fluorescein, including QY, ε and λ_ex_/λ_em_, further proving their assertion that the carboxyl group was not essential. Moreover, replacement of the carboxyl group with a proton thus removing the rotational barrier yielded a non-fluorescent molecule, as predicted. This was believed to be due to the excited molecule now being able to depopulate its electronically excited state thermally, through propeller like rotation of the upper ring^15^.

Urano *et al*. also discovered that the QY of the Tokyo Greens varied with upper ring electron density, and this was determined by functional group substitution of the upper phenyl ring. Their findings demonstrated that as electron density of the upper ring increased, as determined by molecular modelling but also colloquially, a concurrent decrease in QY was observed. This was believed to be as the result of an increase in fluorescence quenching through photo induced electron transfer (PeT), which acted to depopulate the excited electronic state non radiatively. As the Singapore Greens are structural analogues of the of the Tokyo Greens, we sought to determine if the same quenching mechanism was active using their proven method. Following measurement of QY, ε and λ_ex_/λ_em_ of Singapore Greens (**15**) – (**23**) (Table 3), we sought now to ascertain whether this was indeed the case.

**Table 3:** Fluorescence and photophysical properties of the Singapore Green Fluorophores (**15**) – (**23**), all measurements performed in absolute EtOH.

Fluorophore	QY	Ɛ (M^−1^cm^−1^)	λ_ex_ (nm)	λ_em_ (nm)
15	0.647	46044	492	519
16	0.561	25695	499	526
17	0.631	26450	491	519
18	0.476	44527	492	526
19	0.0147	35411	501	527
20	0.391	22822	497	532
21	0.00983	38279	505	525
22	0.564	36186	496	526
23	0.653	17178	499	524

**Figure 2:**
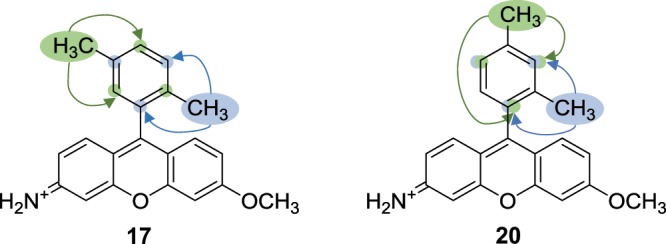
Opposing inductive effects are observed for the CH_3_ groups of (**17**) as a result of their disposition. Conversely, in (**20**) an additive inductive effect is seen, thus increasing electron density in positions indicated. A disproportionate increase in PeT for (**20**) is the result, producing a correspondingly large drop in QY for (**20**).

Interestingly, the two di-methyl analogues, (**17**) and (**20**) were found to exhibit markedly differing quantum yields of 0.63 and 0.39 respectively, despite differing only by disposition of the methyl groups on their upper rings. For (**20**) both methyl groups inductively donate electron density to the same three positions, one of which links the upper ring to the lower xanthene system (Fig. 2). As PeT is somewhat analogous to an ionisation process, and both methyl groups inductively donate electron density to this junction, ionisation at this position is conceivably facilitated. With this effect in mind, it is plausible that ionisation occurs here more readily, resulting in a disproportionate increase in the rate of PeT to the xanthene system, thus quenching fluorescence more significantly than expected. This same effect is not observed for (**17**) due to the para disposition of its methyl groups, in this case both oppose one another’s inductive effects to any specific ring position, nullifying the enhancement to PeT seen with (**20**).

**Figure 3:**
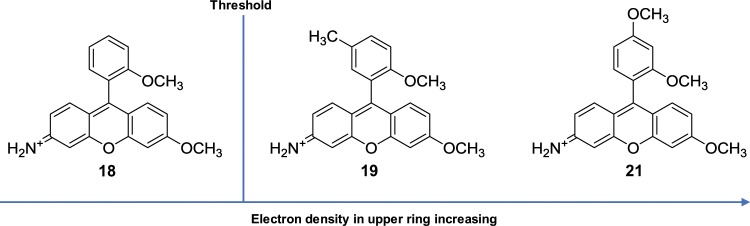
Comparative QYs of (**18**), (**19**) and (**21**). A large drop is seen between (**18**) and (**19**) despite only a modest increase in upper ring electron density. This is indicative of a PeT threshold above which it becomes the dominant depopulator.

Remarkably, there is a huge difference in quantum yield between the mono-methoxy substituted (**18**), and the methoxymethyl substituted (**19**), with quantum yields of 0.48 and 0.015 respectively. This was a surprise to us and suggests that there exists an electron density threshold. Above this PeT is the dominant depopulator of the excited electronic state, at which point QY drops tremendously. Naturally (**21**) possessing two methoxy groups rendering it more electron rich, places it above the PeT threshold and bestows it with a negligible QY of 0.0098 (Fig. 3).

**Figure 4:**
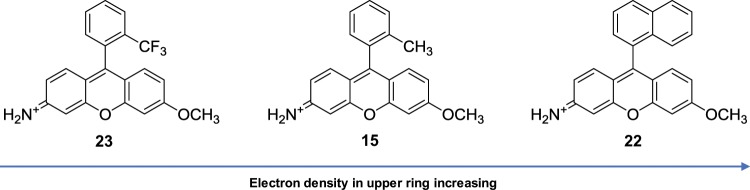
QY displays an inverse proportionality to upper ring electron density.

The three most fluorescent of the Singapore Greens (**15**), (**22**), and (**23**) also follow suit in that their relative upper ring electron densities exhibit the expected inverse proportionality to QY. The least electron rich CF_3_ variant (**23**) being most fluorescent, with the conceivably more electron rich naphthyl substituted (**22**) least fluorescent of the three, with (**15**) in between (Fig. 4).

Considering these observations, we may conclude that a similar PeT quenching mechanism applies to Singapore Greens as it does to Tokyo Greens. This relationship is not in direct proportionality to electron density, but there lies a threshold after which PeT dominates and fluorescence rapidly drops. It may be possible to further assess the PeT threshold more precisely *in silico* so as to determine how substitution of upper ring substituents vary its electron density. This may provide a more robust basis from which to tailor the QY of further novel analogues^16–18^. Unsurprisingly, mesomeric donating effects appear dominant over those observed from inductive. This dominance is similar to that seen in electrophilic aromatic substitution caused by resonance stabilisation of positive charge brought about following PeT ionisation.

Furthermore, as with the Tokyo Greens we have demonstrated that substitution of the upper carboxyl group with a variety substituents has little effect on the λ_ex_/λ_em_^15^. Only slight variations are noted on these properties, which could be attributed to experimental and equipment-based imperfections. The similarity in λ_ex_/λ_em_ to fluorescein and rhodamine 110 is useful, as the industry standard 488 nm argon ion laser is compatible with all of them.

The measured values for the molar extinction coefficient (ε) at first glance appear to show a modest correlation to molecular cross-sectional area. However, (**15**) exhibits the highest extinction coefficient, which is difficult to equate to it possessing a larger molecular cross-sectional area than the naphthyl derivative (**22**), which has a lower coefficient. Therefore, the only conclusion that can be drawn is that other steric, electronic, solvatochromic or agglomeration effects account for the variations seen, and these cannot be sufficiently addressed herein.

### Synthesis of quenched analogues

Acylation was now undertaken on three candidates (**15**), (**19**) and (**22**) in order to generate comparative QYs as proof of concept of their pro-fluorescent nature. This class of fluorophore, if effectively quenched *via* NH_2_ acylation would benefit from achieving maximum fluorescence after only a single cleavage event, unlike probes generated from symmetrical fluorophores which require two.

**Scheme 4:**
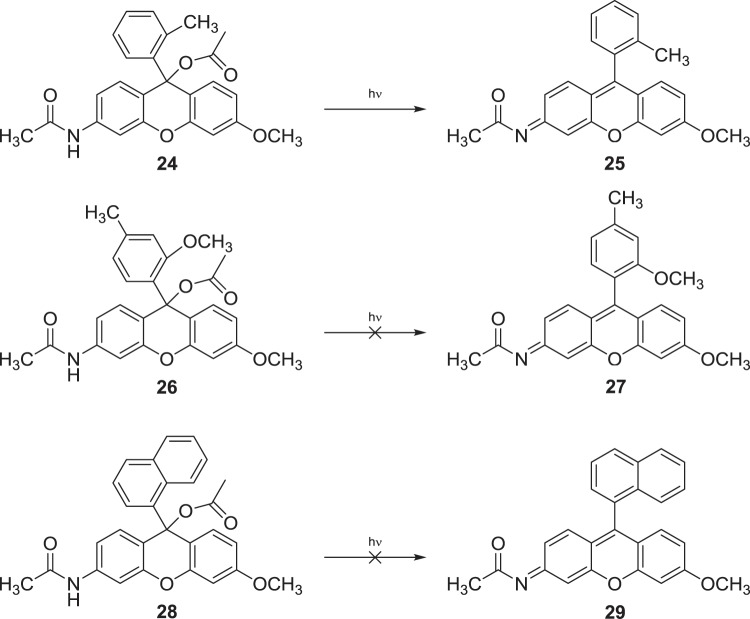
Acylation produced bis acyl species in all cases. Molecule (**24**) was noted to rapidly photolyze, while (**26**) and (**28**) were found to be photolytically stable.

**Figure 5:**
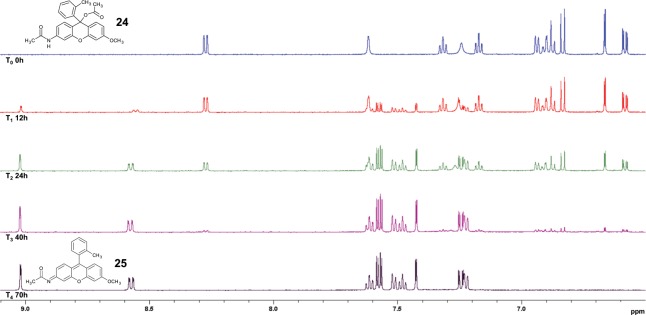
Time lapse ^1^H-NMR of the photolysis of (**24**) into (**25**) (aromatic region).

Standard acylation procedures were employed using excess acetic anhydride in neat anhydrous pyridine. To our surprise, in all cases acylation happened in two positions, producing bis acyl esters (**24**), (**26**) and (**28**) (Scheme 4). Interestingly, bis acyl (**24**) was found to have photolyzed fully in daylight after 70 hours to form the target mono acyl species (**25**) with simultaneous liberation of acetic acid, observed at around 2.02 ppm (Figure S1, Supplemental). Concurrent disappearance of the amide proton at 7.56 ppm (Fig. 5) was also observed. Strangely however, (**26**) and (**28**) were found to be photolytically stable, even to extended periods of UV irradiation at both 365 and 254 nm. Their apparent photostability is postulated to be due to some manner of steric or orientational stabilisation, thereby inhibiting adoption of the correct orientation for decomposition *via* elimination of acetate.

As (**24**) decomposed into the target (**25**), the comparative fluorescence properties were measurable. Acylation had produced an approximate 1100-fold decrease in brightness, easily sufficient for production of a high contrast fluorescent protease probe, proving their pro-fluorescent nature and utility for probe generation (Table 4).

**Table 4:** Comparative fluorescence and photophysical properties of (**15**) and (**25**), measurements performed in absolute EtOH.

Fluorophore	QY (Φ)	ε (M^−1^cm^−1^)	Brightness (Φ × ε)
15	0.647	46044	29810.35
25	0.121	225	27.20

### Biochemical experiments to screen for proteolytic cleavage

A representative compound (**25**) was selected to be assessed as a prospective protease substrate. As this compound had been proven to show significant fluorescence quenching upon NH_2_ acylation (Table 2), proteolytic cleavage of the acyl group to release the free fluorophore (**15**) would demonstrate its applicability as a protease probe.

In order to assess the viability of (**25**) as a protease probe, a variety of protease enzymes were selected for it to be screened against. The proteases tested were as follows: Pepsin, Subtilisin from *Bacillus licheniformis, Streptomyces griseus* protease, Carboxypeptidase A, Papain, Proteinase K and α-chymotrypsin. This selection was deemed sufficiently diverse to determine whether the acyl side chain could be hydrolysed proteolytically. It would also demonstrate that this event does indeed give rise to a fluorescence profile analogous to that of the unacetylated Singapore Green derivative (**15**). These assays were performed in aqueous conditions in the required environment for protease activity in order to simulate the biological system in which the probe would be utilised, (Table 4) The acyl group was found to be rapidly cleaved from (**25**) by both Papain and subtilisin *B. licheniformis* successfully liberating (**15**) to produce a fluorescence profile resembling that observed in absolute EtOH. The proteolytic cleavage of (**25**) providing strong evidence towards the viability of the Singapore Greens as protease probes. Selectivity was also observed towards the cleavage as the other five proteases exposed showed no proteolytic activity whatsoever toward the acylated substrate even after a 24-hour period. It may be inferred from this observation that attachment of a specifically designed polypeptide side chain could facilitate improved selectivity toward specific proteases of interest to produce a fluorescent response. Furthermore, the total lack of hydrolysis of (**25**) observed in the blank after 24 hours exposed to the acidic conditions (~pH 2) in which pepsin is active highlights its excellent stability. This broadens the range of physiological conditions probes constructed from it could tolerate while maintaining selectivity.

**Table 5:** List of enzymes screened with their respective solvent systems and activation conditions.

Protease	Pepsin	Subtilisin *B. licheniformis*	*S. griseus*	Carboxy-Peptidase	Papain	Protease K	α-Chymo-trypsin
Concentration	22.9 mM	148 mM	4.44 mM	11.4 mM	171 mM	14.0 mM	16.0 mM
Solvent system	dd H_2_O 5 mM HCl (pH 2)	dd H_2_O	10 mM NaOAc	Used as supplied	dd H_2_O 1.1 mM EDTA, 0.67 mM EtSH, 5.5 mM cysteine-HCl.	dd H_2_O	0.08 M Tris-HCl (pH 7)

Upon cleavage of (**25**) to release (**15**) the λ_ex_ maximum was noted to have hypsochromically shifted to around 450 nm and become more diffuse, on the other hand the λ_em_ maximum showed only a small blue shift of around 8 nm with a large increase in fluorescence intensity. Examination of the λ_ex_/λ_em_ spectrum of (**15**) post cleavage in PBS (see supplemental section) provides good evidence toward its applicability for production of physiological protease probes. See both Figure S40 (Supplemental section) for protease assay, and Table 5 for assay conditions.

## Methods

### General methods

All ^1^H-NMR spectra were recorded at either 400 MHz on a Brüker Avance AV400 FT-NMR spectrometer or on a 600 MHz Brüker Avance AV600. ^13^C-NMR spectra were recorded at 100 MHz using the Brüker AV400 or at 125 MHz using the Brüker AV600. ^19^F spectra were obtained at 376 MHz on the Brüker AV400. NMR spectral measurements were carried out in solution using deuterated solvents: CDCl_3_, DCM, DMSO, or MeOD as the solvent. NMR signals quoted are in ppm as downfield δ chemical shifts from either the internal tetramethylsilane (TMS) standard at δ = 0.00 or from the residual CHCl_3_ proton resonance at δ = 7.26 ppm, residual DCM resonance at δ = 5.32, residual DMSO resonance at δ = 2.50 ppm, or the residual MeOD resonance at δ = 3.31 ppm. All coupling constants are quoted in Hz. TLC analysis was performed using Fluka glass backed silica gel plates 60 F_254_, 250 μm or Merck aluminium backed aluminium oxide 60 F_254_. Flash column chromatography was performed at ambient temperature using high purity Merck 63–200 μm silica gel at moderate pressure, or with a Biotage Isolera Spektra Four system using Biotage ZIP 40–60 μm flash cartridges. Low resolution mass spectra (LR MS): EI (electron impact); ES (electro spray); CI (chemical ionisation) and High-resolution mass spectra (HR MS) were obtained *via* MEDAC Ltd. in accordance with BS EN ISO 9001:2008 provision for microanalysis service. Infra-red analysis was performed using either neat oil or a solid powdered sample coated onto the analysis plate of a ThermoScientific Nicolet 869–142500 iD5 Diamond ATR FT-IR spectrophotometer, or coated onto either a KBr or NaCl disc and analysed using a ThermoScientific Nicolet iS5 FT-IR spectrophotometer. UV-Vis spectrophotometry was performed using an Agilent Cary 7000 UMS spectrophotometer using ultra-pure optically matched, stoppered Spectrosil quartz fluorimeter cells, type 23-Q-10 with a 10 mm path length. Scan rate of 600 nm/min was employed, all spectral measurements were made in absolute EtOH unless otherwise stated. Fluorescence measurement was performed using an Agilent Cary Eclipse fluorescence spectrophotometer using the same fluorimeter cells, all fluorescence measurements were made in absolute EtOH unless otherwise stated. All chemicals were purchased from commercial vendor Sigma-Aldrich chemicals unless otherwise stated and purchased at the highest possible grade (at least > 95%) and used as received. All anhydrous solvents were also purchased at the highest possible grade from the same vendor, obtained in SureSeal packaging and stored under nitrogen at 4 °C. Other solvents were of SLR grade from the same vendor. Anhydrous and air free reactions were performed using oven then flame dried glassware under vacuum, and carried out under an anhydrous nitrogen atmosphere. Where no reaction temperature is given, reactions were carried out at an ambient temperature of 20 °C.

### Biochemical methods

All protease enzymes were purchased from commercial vendor Sigma-Aldrich chemicals, used as supplied and prepared according to standard methodology (Table 3) A solution of protease probe (**25**) was freshly prepared using 1.50 mg (**25**) SG1Ac dissolved in 50 µl absolute EtOH and diluted to a total volume of 1120 µl using PBS at pH 7.4. Absorbance and emission maxima for SG1Ac was measured in the same PBS based solvent system. To assess the proteolytic cleavage of compound (**25**), a comparative assay using a library of 7 different proteases was performed (Table 3). A 96 well plate format was used, equal concentrations (1.5 mM) of compound (**25**) were prepared for each of the different enzyme solutions at the concentrations stated in Table 3 and diluted to a final volume of 50 µl. To accommodate for the potential effects the different enzyme activation solutions/solvents systems could have on compound (**25**), controls were used (referred to as Blank in Fig. 5) containing the respective enzyme medium only (in the absence of enzyme). Emitted fluorescent readouts were recorded over time at controlled room temperature environment using a Tecan Infinite M200 Pro microplate reader. At the end of the assay excitation and emission spectra before and after cleavage were recorded for comparison. Data was plotted using GraphPad Prism 8 (Graph Pad Software Inc., San Diego, CA), (Supplemental Figure S40).

## Synthetic Methods

### 3-Amino-6-nitro-xanthen-9-one, (1)

To a stirred suspension of 3-acetamidophenol (18.10 g, 120 mmol) in anhydrous DMF (200 ml) under a dry nitrogen atmosphere, was added anhydrous K_2_CO_3_ (16.60 g, 120 mmol), 2-chloro-4-nitrobenzoic acid and copper powder (0.40 g, 6.00 mmol). The resulting mixture was heated to 130 °C for 16 hours, then cooled to room temperature. The crude mixture was quenched by pouring over 500 ml of iced 5 M HCl solution and adjusted to pH 1 *via* further HCl addition. The mixture was stirred until a pale brown solid had precipitated, the solid was then isolated, washed with cold water and dried in air. The solid was then slowly dissolved in concentrated H_2_SO_4_ (100 ml) and heated to 80 °C for 1 hour with stirring. After cooling to room temperature, the mixture was poured over ice (300 ml) and stirred until a brown solid formed, the solid was isolated and stirred with 20% w/v Na_2_CO_3_ until evolution of CO_2_ ceased. The crude product was washed with water and dried under vacuum. Purification was achieved *via* successive reverse recrystallisation and decantation using ethyl acetate:petroleum ether (40–60) 1:1 to pure ethyl acetate. The pure product was isolated as a bright yellow powder. (9.220 g, 30%, 1:1 ethyl acetate:petroleum ether (40–60) R_f_ = 0.2). ^1^H-NMR (400 MHz; d^6^ - DMSO) δ = 6.56 (1 H, d, ^4^*J*_H,H_ = 2.04 Hz, Ar-H), 6.70 (2 H, s, NH_2_), 6.71 (1 H, dd, ^4^*J*_H,H_ = 2.04, ^3^*J*_H,H_ = 8.79 Hz, Ar-H), 7.87 (1 H, d, ^3^*J*_H,H_ = 8.79 Hz, Ar-H), 8.14 (1 H, dd, ^4^*J*_H,H_ = 2.14, ^3^*J*_H,H_ = 8.65 Hz, Ar-H), 8.31 (1 H, d, ^3^*J*_H,H_ = 8.65 Hz, Ar-H), 8.34 (1 H, d, ^4^*J*_H,H_ = 2.14 Hz, Ar-H). ^13^C-NMR (100 MHz; d^6^ - DMSO) δ = 97.33, 110.67, 113.15, 113.49, 117.76, 125.69, 127.60, 127.71, 150.23, 154.81, 156.46, 158.32, 172.38. IR (ATR): v = 3479 (m), 3345 (m) cm^−1^ (NH_2_), 1646 (m) cm^−1^ (C=O). LR MS (EI) m/z = found 257.1, requires 257.1 [M + H]^+^.

### 3-Hydroxy-6-nitro-xanthen-9-one, (2)

To a stirred solution of 3-amino-6-nitro-xanthen-9-one (1.48 g, 5.78 mmol) in a mixture of concentrated H_2_SO_4_ (30 ml) and water (15 ml) at 0 °C, a solution of NaNO_2_ (1.20 g, 17.3 mmol) in water (10 ml) was added dropwise. The mixture was stirred for 1 hour at 0 °C, then poured into boiling water (1000 ml) and held at 90 °C for a 1 hour. The reaction was cooled to room temperature, the solid isolated, washed with excess cold water and dried under vacuum to yield the pure product as a light brown powder. (1.283 g, 86%). ^1^H-NMR (400 MHz; d^6^ - DMSO) δ = 6.88 (1 H, d, ^4^*J*_H,H_ = 2.20 Hz, Ar-H), 6.94 (1 H, dd, ^4^*J*_H,H_ = 2.20, ^3^*J*_H,H_ = 8.75 Hz, Ar-H), 8.03 (1 H, d, ^3^*J*_H,H_ = 8.75 Hz, Ar-H), 8.15 (1 H, dd, ^4^*J*_H,H_ = 2.16, ^3^*J*_H,H_ = 8.67 Hz, Ar-H), 8.33 (1 H, d, ^3^*J*_H,H_ = 8.67 Hz, Ar-H), 8.36 (1 H, d, ^4^*J*_H,H_ = 2.16 Hz, Ar-H), 11.12 (1 H, s, OH). ^13^C-NMR (100 MHz; d^6^ - DMSO) δ = 102.16, 113.77, 113.92, 114.85, 118.05, 125.13, 127.85, 128.11, 150.57, 154.98, 157.85, 164.65, 173.63. IR (ATR): v = 3074 (s) cm^−1^ (OH), 1649 (m) cm^−1^ (C=O). LR MS (EI) m/z = found 256.0, requires 256.0 [M − H]^+^.

### 3-Methoxy-6-nitro-xanthen-9-one, (3)

To a suspension of 3-hydroxy-6-nitro-xanthen-9-one (1.28 g, 4.98 mmol) in anhydrous DMF (30 ml) under a dry nitrogen atmosphere, was added anhydrous K_2_CO_3_ (1.38 g, 9.98 mmol). The suspension was stirred for 20 minutes, after which iodomethane (0.850 g, 5.99 mmol) was added dropwise. The resulting mixture was stirred at room temperature for 18 hours, after which TLC analysis showed complete disappearance of starting material. The crude reaction mixture was diluted with chloroform (30 ml), filtered, the residue washed several times with chloroform and the organic extracts combined. The organic extracts were washed with saturated aqueous LiCl solution (3 ×50 ml), and the LiCl layers back extracted with chloroform. The organics were combined, washed with saturated aqueous NaHCO_3_ (3 ×50 ml), water (2 ×50 ml) and brine (2 ×50 ml). The washing process was repeated until the organic layer was clean *via* TLC, after which it was dried over Na_2_SO_4_ followed by vacuum, to yield a beige powder. (1.086 g, 80%). ^1^H-NMR (400 MHz; d^6^ - DMSO) δ = 3.98 (3 H, s, CH_3_), 6.95 (1 H, d, ^4^*J*_H,H_ = 2.36 Hz, Ar-H), 7.02 (1 H, dd, ^4^*J*_H,H_ = 2.36, ^3^*J*_H,H_ = 8.91 Hz, Ar-H), 8.18 (1 H, dd, ^4^*J*_H,H_ = 2.12, ^3^*J*_H,H_ = 8.67 Hz, Ar-H), 8.27 (1 H, d, ^3^*J*_H,H_ = 8.91 Hz, Ar-H), 8.36 (1 H, dd, ^5^*J*_H,H_ = 0.32, ^4^*J*_H,H_ = 2.12 Hz, Ar-H), 8.50 (1 H, dd, ^5^*J*_H,H_ = 0.32, ^3^*J*_H,H_ = 8.67 Hz, Ar-H). ^13^C-NMR (100 MHz; d^6^ - DMSO) δ = 56.27, 100.69, 113.79, 114.36, 114.95, 118.28, 125.17, 127.73, 127.96, 150.75, 155.09, 157.92, 165.51, 173.91. IR (ATR): v = 3104 (m) cm^−1^ (Ar-CH), 1668 (s) cm^−1^ (C=O). LR MS (EI) m/z = found 271.0, requires 271.1 [M]^+^.

### 3-Methoxy-6-(tritylamino)xanthen-9-one, (4)

To a solution of 3-amino-6-methoxy-xanthen-9-one (0.94 g, 3.90 mmol) in anhydrous DCM (60 ml), was added triethylamine (1.58 g, 2.18 ml, 15.6 mmol) followed by trityl chloride (2.17 g, 7.80 mmol). The reaction was stirred at room temperature whilemonitoring *via* TLC analysis, complete consumption of the starting material was noted after 1 hour. The crude reaction mixture was then diluted with water (50 ml), the aqueous portion separated and successively washed with DCM (3 ×50 ml). The organics were then combined, washed with water (3 ×50 ml), brine (2 ×50 ml), dried over anhydrous Na_2_SO_4_ and the solvent removed under vacuum. Purification was achieved *via* dissolution in DCM and subsequent precipitation in excess vigorously stirred n-hexane. The product was isolated, washed several times with 10:1 to 20:1 n-hexane:ethyl acetate and dried under vacuum to yield a very light yellow powder. (1.523 g, 81%). ^1^H-NMR (400 MHz; CDCl_3_) δ = 3.85 (3 H, s, CH_3_), 5.66 (2 H, s, NH), 6.07 (1 H, d, ^4^*J*_H,H_ = 2.26 Hz, Ar-H), 6.54 (1 H, dd, ^4^*J*_H,H_ = 2.26, ^3^*J*_H,H_ = 8.77 Hz, Ar-H), 6.69 (1 H, d, ^4^*J*_H,H_ = 2.44 Hz, Ar-H), 6.84 (1 H, dd, ^4^*J*_H,H_ = 2.44, ^3^*J*_H,H_ = 8.87 Hz, Ar-H), 7.94 (1 H, d, ^3^*J*_H,H_ = 8.77 Hz, Ar-H), 8.14 (1 H, d, ^3^*J*_H,H_ = 8.87 Hz, Ar-H). ^13^C-NMR (100 MHz; CDCl_3_) δ = 55.82, 71.92, 100.22, 101.45, 112.53, 113.16, 114.03, 116.09, 127.09, 127.47, 128.03, 128.41, 129.21, 144.45, 151.93, 157.62, 157.92, 164.30, 175.40. IR (ATR): v = 3316 (m) cm^−1^ (NH), 1650 (w) cm^−1^ (C=O). HR MS (ES) m/z = found 484.1925, requires 484.1913 [M + H]^+^.

### 2-Chlorotrityl resin bound 3-Methoxy-6-(tritylamino)xanthen-9-one, (5)

The 2-chlorotrityl chloride resin (2.60 g, 4.16 mmol trityl sites) was swelled in anhydrous DCM (50 ml) under a nitrogen atmosphere for 30 minutes, the solvent was removed and the resin rinsed with anhydrous DCM (2 ×10 ml). The resin was then dried under a stream of nitrogen, after which a pyridine (3.29 g, 41.6 mmol, 3.36 ml) and a solution of 3-methoxy-6-(tritylamino)xanthen-9-one (1.00 g, 4.16 mmol) in anhydrous DCM/DMF (50 ml/10 ml) was added. The resulting suspension was agitated for 36 hours, after which the solution was removed under positive nitrogen pressure. The crude resin was washed with anhydrous DMF (5 ×30 ml), anhydrous methanol (10 ×20 ml) and anhydrous DCM (10 × 20 ml), then dried under a nitrogen stream. A solution of DCM:Methanol:DIEA (34 ml:4 ml:2 ml) was then added to the resin under a dry nitrogen atmosphere and the resulting suspension was agitated for 1 hour at room temperature. The capping solution was then removed under positive nitrogen pressure and washed with DCM (3 ×20 ml), DMF (3 ×20 ml), methanol (3 ×20 ml) and DCM (3 ×20 ml), dried under a nitrogen stream, vacuum desiccated at 50 °C for 48 h, then stored under nitrogen.

### 6-Methoxy-9-(o-tolyl)xanthen-3-iminium trifluoroacetate (15)

Into a flask containing magnesium turnings (0.13 g, 5.15 mmol) under a dry nitrogen atmosphere was added anhydrous diethyl ether (7.7 ml). Into this was added a solution 1-bromo-2-methyl benzene (0.88 g, 5.15 mmol, 0.89 ml) in anhydrous THF (2.6 ml) dropwise, with stirring and heating to mild reflux. Upon Grignard initiation, heating was removed and the remaining bromide continued to be added dropwise with stirring to maintain a mild reflux until all bromide was added. The mixture was stirred for a further 1 h at which point a solution of 3-methoxy-6-(tritylamino)xanthen-9-one (0.50 g, 1.03 mmol) in anhydrous THF (10 ml) was added dropwise over 30 minutes, followed by heating to 60 °C. The mixture was maintained at 60 °C for 16 hours and followed by TLC analysis, after which a second portion of Grignard reagent was added, prepared in the same manner as before using magnesium (0.13 g, 5.15 mmol) and bromo-2-methyl benzene (1.04 g, 2.06 mmol, 1.03 ml) in anhydrous THF (4 ml). Following addition, the mixture was stirred for a further 18 h at 60 °C and followed by TLC analysis. After 36 h the reaction was quenched with water (10 ml), stirred for 30 min and slowly acidified to pH 7 with cold aqueous 1 M HCl. Subsequently the solution was extracted with diethyl ether (3 ×10 ml) and ethyl acetate (1 ×10 ml), the organics combined and washed with water (2 ×10 ml) and brine (1 ×10 ml), then dried over anhydrous Na_2_SO_4_ and concentrated under vacuum. The crude product was reconstituted in DCM (14 ml), to which water (4 ml) and trifluoroacetic acid (2 ml) were added with stirring at room temperature. The mixture was stirred for 30 minutes after which water (10 ml) was added, and the organic layer separated. The organics were washed with saturated NaHCO_3_ (2 ×10 ml), water (1 ×10 ml) and brine (1 ×10 ml), then dried over anhydrous Na_2_SO_4_ and concentrated under vacuum. Purification was achieved *via* flash column chromatography using a gradient elution of 0–10% methanol:DCM, to yield the product as a foamy red solid. (0.035 g, 10.9%, 1:99 methanol:DCM R_f_ = 0.1). ^1^H-NMR (600 MHz; MeOD) δ = 2.05 (3 H, s, CH_3_), 4.08 (3 H, s, CH_3_), 7.00 (1 H, d, ^4^*J*_H,H_ = 1.92 Hz, Ar-H), 7.07 (1 H, dd, ^4^*J*_H,H_ = 1.92, ^3^*J*_H,H_ = 9.36 Hz, Ar-H), 7.17 (1 H, dd, ^4^*J*_H,H_ = 2.34, ^3^*J*_H,H_ = 9.12 Hz, Ar-H), 7.28 (1 H, d, ^3^*J*_H,H_ = 7.50 Hz Ar-H), 7.31 (1 H, d, ^3^*J*_H,H_ = 9.36 Hz, Ar-H), 7.34 (1 H, d, ^3^*J*_H,H_ = 9.12 Hz, Ar-H), 7.45 (1 H, d, ^4^*J*_H,H_ = 2.34 Hz, Ar-H), 7.49 (1 H, dd, ^3^*J*_H,H_ = 7.50, ^3^*J*_H,H_ = 7.50 Hz, Ar-H), 7.54 (1 H, d, ^3^*J*_H,H_ = 7.68 Hz, Ar-H), 7.60 (1 H, dd, ^3^*J*_H,H_ = 7.50, ^3^*J*_H,H_ = 7.50 Hz, Ar-H), 8.72 (2 H, br, NH_2_). ^13^C-NMR (150 MHz; MeOD) δ = 19.66, 57.57, 98.56, 98.63, 101.55, 116.65, 118.29, 121.31, 121.38, 127.37, 130.07, 131.63, 131.98, 132.27, 132.71, 134.34, 137.21, 158.49, 161.51, 161.73, 164.19, 164.30, 169.67. ^19^F-NMR (376 MHz; MeOD) δ = −76.77. IR (ATR): v = 3050 (m) cm^−1^ (NH_2_^+^), 1645 (m) cm^−1^ (NH_2_^+^). HR MS (ES) m/z = found 316.1341, calculated 316.1338 [M]^+^.

### 6-Methoxy-9-[2-(trifluoromethyl)phenyl]xanthen-3-iminium hydrochloride (16)

Into a flask containing magnesium turnings (0.16 g, 6.51 mmol) under a dry nitrogen atmosphere was added anhydrous THF (6 ml). Into this, a solution of 2-bromobenzotrifluoride (1.47 g, 6.51 mmol, 0.89 ml) in anhydrous THF (4 ml) was added dropwise, with stirring and heating to mild reflux. Upon Grignard initiation, heating was removed and the remaining bromide continued to be added dropwise with stirring to maintain a mild reflux until all was added. The mixture was then stirred for a further 1 h at which point a solution of 3-methoxy-6-(tritylamino)xanthen-9-one (0.45 g, 0.930 mmol) in anhydrous THF was added dropwise over 30 minutes, followed by heating to 60 °C. The mixture was maintained at 60 °C for 18 h and followed by TLC analysis. After the initial 18 h a second portion of Grignard reagent was added, prepared in the same manner as before using magnesium (0.11 g, 4.65 mmol) and 2-bromobenzotrifluoride (1.05 g, 4.65 mmol, 0.63 ml) in anhydrous THF (4 ml). Following addition, the mixture was stirred for a further 18 h at 60 °C and followed by TLC analysis. After 36 h the reaction was quenched with water (10 ml), stirred for 30 min and slowly acidified to pH 7 with cold aqueous 1 M HCl. Subsequently the solution was extracted with diethyl ether (3 × 10 ml) and ethyl acetate (1 × 10 ml), the organics combined and washed with water (2 × 10 ml) and brine (1 × 10 ml), then dried over anhydrous Na_2_SO_4_ and concentrated under vacuum. The crude product was reconstituted in THF (30 ml), to which 6 M aqueous HCl (30 ml) was added. The solution was stirred and followed by TLC analysis and after 1 h the deprotection was deemed complete. Saturated NaHCO_3_ solution (30 ml) was then added to the resulting mixture with stirring until CO_2_ evolution ceased. The mixture was basified to pH 7 with 10% aqueous NaOH. The mixture was then extracted with DCM (5 × 20 ml), the organics washed with water (3 × 10 ml) and brine (1 × 20 ml), then dried over anhydrous Na_2_SO_4_ and concentrated under vacuum. Purification was achieved *via* flash column chromatography using a gradient elution 0–10% Methanol:DCM to yield a fluffy red solid. (0.050 g, 13.2%, 1:99 methanol:DCM R_f_ = 0.1). ^1^H-NMR (600 MHz; MeOD) δ = 4.08 (3 H, s, CH_3_), 7.00 (1 H, d, ^4^*J*_H,H_ = 2.04 Hz, Ar-H), 7.07 (1 H, dd, ^4^*J*_H,H_ = 2.04, ^3^*J*_H,H_ = 9.36 Hz, Ar-H), 7.16 (1 H, dd, ^4^*J*_H,H_ = 2.40, ^3^*J*_H,H_ = 9.12 Hz, Ar-H), 7.23 (2 H, d, ^3^*J*_H,H_ = 9.36 Hz, Ar-H), 7.24 (1 H, d, ^3^*J*_H,H_ = 9.12 Hz, Ar-H), 7.47 (1 H, d, ^3^*J*_H,H_ = 2.40 Hz, Ar-H), 7.54–7.56 (1 H, m, Ar-H), 7.90–7.95 (2 H, m, Ar-H), 8.06–8.08 (1 H, m, Ar-H). ^13^C-NMR (150 MHz; MeOD) δ = 57.62, 98.57, 101.42, 117.00, 118.25, 118.80, 121.49, 128.15, 129.99, 130.19, 131.24, 132.02, 132.20, 132.34, 133.92, 134.49, 158.13, 158.19, 161.29, 164.45, 169.72. ^19^F-NMR (376 MHz; MeOD) δ = −60.12 (CF_3_). IR (ATR): v = 3400 (s) cm^−1^ (NH_2_^+^), 1649 (m) cm^−1^ (NH_2_^+^). HR MS (ES) m/z = found 370.1053, calculated 370.1055 [M]^+^.

### 9-(2,5-Dimethylphenyl)-6-methoxy-xanthen-3-iminium trifluoroacetate (17)

Into a flask containing magnesium turnings (0.23 g, 9.49 mmol) under a dry nitrogen atmosphere was added anhydrous THF (4 ml). Into this a solution of 2-bromo-1,4-dimethyl benzene (1.69 g, 9.11 mmol, 1.26 ml) in anhydrous THF (4 ml) was added dropwise, with stirring and heating to mild reflux. Upon Grignard initiation, heating was removed and the remaining bromide continued to be added dropwise over 30 minutes with stirring to maintain a mild reflux until all bromide was added, the reaction was then stirred for a further 1 h at room temperature. The 2-chlorotrityl chloride resin bound 3-methoxy-6-(tritylamino) xanthen-9-one (0.27 mg/mg loading by qNMR, 0.69 g resin, 0.18 g loading, 0.759 mmol) was swelled in anhydrous THF (10 ml) for 30 minutes prior to use, the solvent was then removed, after which the Grignard reagent was added and heated to 60 °C. The reaction was agitated and maintained at 60 °C for 24 h while following *via* TLC analysis. After the initial 16 h the reaction solvent was removed and a second portion of Grignard reagent was added (12 equivalents), prepared in the same manner as before. Again, this was agitated at 60 °C for a further 16 h, after which the reaction mixture was removed and a third portion of Grignard (12 equivalents) was added as before and agitated at 60 °C for 16 h. Following this, the reaction mixture was cooled to room temperature, removed, and the resin washed with DMF (5 × 10 ml), methanol (10 × 10 ml), DCM (10 × 10 ml) and diethyl ether (5 × 10 ml), then dried under a stream of nitrogen and vacuum desiccated. The resin was then stirred in a 5% trifluoroacetic acid solution in DCM (1 ml/20 ml) for 20 minutes, the solution was removed, retained and the resin further washed with 5% trifluoroacetic acid in DCM (3 × 20 ml). The extracts were combined and concentrated under vacuum. Purification was achieved *via* flash column chromatography using a gradient elution of 0–10% methanol:DCM, to yield a red foam. (0.061 g, 18.1%, 1:99 methanol:DCM R_f_ = 0.1). ^1^H-NMR (600 MHz; CD_2_Cl_2_) δ = 1.96 (3 H, s, CH_3_), 2.39 (3 H, s, CH_3_), 4.01 (3 H, s, CH_3_), 6.98 (1 H, br s, Ar-H), 7.00 (1 H, dd, ^4^*J*_H,H_ = 2.46, ^3^*J*_H,H_ = 9.06 Hz, CH-8), 7.14 (1 H, br s, Ar-H), 7.15 (1 H, dd, ^4^*J*_H,H_ = 1.80, ^3^*J*_H,H_ = 9.06 Hz, Ar-H), 7.21 (1 H, d, ^4^*J*_H,H_ = 2.46 Hz, Ar-H), 7.23 (1 H, d, ^3^*J*_H,H_ = 9.06 Hz, Ar-H), 7.27 (1 H, d, ^3^*J*_H,H_ = 9.06 Hz, Ar-H), 7.32–7.35 (2 H, m, Ar-H), 8.93 (2 H, br, NH_2_). ^13^C-NMR (150 MHz; CD_2_Cl_2_) δ = 19.31, 21.02, 57.26, 57.56, 98.70, 100.53, 100.73, 115.85, 117.56, 117.65, 121.05, 129.59, 131.16, 131.39, 131.58, 131.65, 133.17, 133.58, 136.51, 157.45, 160.23, 161.22, 162.56, 168.39. ^19^F-NMR (376 MHz; CD_2_Cl_2_) δ = −75.93. IR (ATR): v = 3069 (m) cm^−1^ (NH_2_^+^), 1644 (m) cm^−1^ (NH_2_^+^). HR MS (ES) m/z = found 330.1507, calculated 330.1494 [M]^+^.

All subsequent Grignard reactions were performed using the same solid supported procedure, the crude product cleaved from the resins using the same method. Stoichiometries for preparation are quoted.

### 6-Methoxy-9-(2-methoxyphenyl)xanthen-3-iminium trifluoroacetate (18)

2-Bromoanisole (2.33 g, 12.4 mmol, 1.55 ml), magnesium turnings (0.32 g, 13.0 mmol) × 3 portions. 2-chlorotritylchloride resin bound 3-methoxy-6-(tritylamino)xanthen-9-one (0.27 mg/mg loading by qNMR, 0.94 g resin, 0.25 g loading, 1.04 mmol). Purification achieved *via* flash column chromatography using gradient elution 2–10% methanol:DCM to yield a foamy red solid. (0.67 g, 14.5%, 1:99 methanol:DCM R_f_ = 0.1). ^1^H-NMR (600 MHz; CD_2_Cl_2_) δ = 3.70 (3 H, s, CH_3_), 4.01 (3 H, s, CH_3_), 6.99 (1 H, dd, ^4^*J*_H,H_ = 2.46, ^3^*J*_H,H_ = 9.12 Hz, Ar-H), 7.17 (1 H, d, ^4^*J*_H,H_ = 2.46 Hz, Ar-H), 7.17 (1 H, br d, ^3^*J*_H,H_ = 8.40 Hz, Ar-H), 7.19–7.20 (2 H, m, Ar-H), 7.30 (1 H, d, ^4^*J*_H,H_ = 1.83 Hz, Ar-H), 7.30 (1 H, d, ^3^*J*_H,H_ = 9.39 Hz, Ar-H), 7.35 (1 H, dd, ^4^*J*_H,H_ = 1.83, ^3^*J*_H,H_ = 9.39 Hz, Ar-H), 7.36 (1 H, d, ^3^*J*_H,H_ = 9.12 Hz, Ar-H), 7.61–7.66 (1 H, m, Ar-H), 9.33 (2 H, br, NH_2_). ^13^C-NMR (150 MHz; CD_2_Cl_2_) δ = 56.05, 57.05, 98.55, 100.34, 112.01, 115.83, 116.61, 118.13, 120.70, 121.20, 121.41, 131.03, 131.46, 132.60, 133.43, 156.93, 157.04, 157.09, 159.95, 163.17, 167.42. ^19^F-NMR (376 MHz; CD_2_Cl_2_) δ = −75.52. IR (ATR): v = 3012 (m) cm^−1^ (NH_2_^+^), 1644 (m) cm^−1^ (NH_2_^+^). HR MS (ES) m/z = found 332.1291, calculated 332.1287 [M]^+^.

### 6-Methoxy-9-(2-methoxy-5-methyl-phenyl)xanthen-3-iminium trifluoroacetate (19)

2-Bromo-4-methylanisole (2.65 g, 13.2 mmol, 1.91 ml), magnesium turnings (0.33 g, 13.7 mmol) × 3 portions. 2-chlorotritylchloride resin bound 3-methoxy-6-(tritylamino) xanthen-9-one (0.27 mg/mg loading by qNMR, 1.00 g resin, 0.26 g loading, 1.10 mmol). Purification achieved *via* flash column chromatography using gradient elution 2–10% methanol:DCM to yield a foamy red solid. (0.158 g, 32.3%, 1:99 methanol:DCM R_f_ = 0.1). ^1^H-NMR (600 MHz; CD_2_Cl_2_) δ = 2.37 (3 H, s, CH_3_), 3.66 (3 H, s, CH_3_), 4.02 (3 H, s, CH_3_), 6.98–6.99 (2 H, m, Ar-H), 7.02 (1 H, dd, ^4^*J*_H,H_ = 2.45, ^3^*J*_H,H_ = 9.04 Hz, Ar-H), 7.05–7.07 (2 H, m, Ar-H), 7.20 (1 H, d, ^4^*J*_H,H_ = 2.45 Hz, Ar-H), 7.37 (1 H, d, ^3^*J*_H,H_ = 9.42 Hz, Ar-H), 7.41 (1 H, d, ^3^*J*_H,H_ = 9.04 Hz, Ar-H), 7.42 (1 H, dd, ^4^*J*_H,H_ = 2.15, ^3^*J*_H,H_ = 9.18 Hz, Ar-H), 8.58 (2 H, br, NH_2_). ^13^C-NMR (150 MHz; CD_2_Cl_2_) δ = 20.47, 56.06, 57.10, 98.12, 100.31, 111.97, 115.95, 116.94, 117.85, 120.27, 120.67, 130.91, 131.25, 131.78, 133.06, 133.91, 154.95, 157.12, 158.35, 160.28, 162.81, 167.80. ^19^F-NMR (376 MHz; CD_2_Cl_2_) δ = −75.97. IR (ATR): v = 3080 (m) cm^−1^ (NH_2_^+^), 1678 (m) cm^−1^ (NH_2_^+^). HR MS (ES) m/z = found 346.1455, calculated 346.1443[M]^+^.

### 9-(2,4-Dimethylphenyl)-6-methoxy-xanthen-3-iminium trifluoroacetate (20)

1-bromo-2,4-dimethylbenzene (1.84 g, 9.94 mmol, 1.34 ml), magnesium turnings (0.25 g, 10.4 mmol) × 3 portions. 2-chlorotritylchloride resin bound 3-methoxy-6-(tritylamino)xanthen −9-one (0.30 mg/mg loading by qNMR, 0.67 g resin, 0.20 g xanthone, 0.829 mmol). Purification achieved *via* flash column chromatography using gradient elution 2–10% methanol:DCM to yield a foamy red solid. (0.135 g, 36.7%, 1:99 methanol:DCM R_f_ = 0.1). ^1^H-NMR (600 MHz; CD_2_Cl_2_) δ = 1.98 (3 H, s, CH_3_), 2.47 (3 H, s, CH_3_), 4.07 (3 H, s, CH_3_), 6.99 (1 H, br s, Ar-H) 7.07 (1 H, d, ^3^*J*_H,H_ = 7.68 Hz, Ar-H), 7.11 (1 H, dd, ^4^*J*_H,H_ = 2.46, ^3^*J*_H,H_ = 9.27 Hz, Ar-H), 7.25 (1 H, br d, ^3^*J*_H,H_ = 7.68 Hz, Ar-H), 7.29 (1 H, d, ^3^*J*_H,H_ = 9.48 Hz, Ar-H), 7.29 (1 H, br s, Ar-H), 7.37 (1 H, d, ^4^*J*_H,H_ = 2.46 Hz, Ar-H), 7.40 (1 H, d, ^3^*J*_H,H_ = 9.27 Hz, Ar-H), 7.69 (1 H, d, ^3^*J*_H,H_ = 9.48 Hz, Ar-H), 11.45 (2 H, br, NH_2_). ^13^C-NMR (150 MHz; CD_2_Cl_2_) δ = 24.53, 26.21, 62.67, 106.42, 122.02, 123.64, 124.22, 125.19, 132.84, 134.44, 135.03, 137.34, 137.52, 137.63, 142.06, 145.13, 147.17, 161.28, 163.73, 164.43, 168.88, 175.38. ^19^F-NMR (376 MHz; CD_2_Cl_2_) δ = −75.88. IR (ATR): v = 3066 (m) cm^−1^ (NH_2_^+^), 1688 (m) cm^−1^ (NH_2_^+^). HR MS (ES) m/z = found 364.1097, calculated 364.1104 [M − H + Cl]^+^.

### 9-(2,4-Dimethoxyphenyl)-6-methoxy-xanthen-3-iminium trifluoroacetate (21)

1-bromo-2,4-dimethoxy benzene (2.16 g, 9.95 mmol, 1.43 ml), magnesium turnings (0.25 g, 10.4 mmol) × 3 portions. 2-chlorotritylchloride resin bound 3-methoxy-6-(tritylamino) xanthen-9-one (0.21 mg/mg loading by qNMR, 0.96 g resin, 0.20 g xanthone loading, 0.829 mmol). Purification achieved *via* flash column chromatography using gradient elution 2–10% methanol:DCM to yield a foamy red solid. (0.68 g, 17.3%, 1:99 methanol:DCM R_f_ = 0.1). ^1^H-NMR (600 MHz; CD_2_Cl_2_) δ = 3.68 (3 H, s, CH_3_), 3.93 (3 H, s, CH_3_), 4.01 (1 H, s, CH_3_), 6.71 (1 H, d, ^4^*J*_H,H_ = 2.22 Hz, Ar-H), 6.74 (1 H, dd, ^4^*J*_H,H_ = 2.22, ^3^*J*_H,H_ = 8.34 Hz, Ar-H), 6.96 (1 H, d, ^4^*J*_H,H_ = 1.83 Hz, Ar-H), 7.03 (1 H, dd, ^4^*J*_H,H_ = 2.46, ^3^*J*_H,H_ = 9.18 Hz, Ar-H), 7.05 (1 H, dd, ^4^*J*_H,H_ = 1.83, ^3^*J*_H,H_ = 9.36 Hz, Ar-H), 7.12 (1 H, d, ^3^*J*_H,H_ = 8.34 Hz, Ar-H), 7.18 (1 H, d, ^4^*J*_H,H_ = 2.46 Hz, Ar-H), 7.42 (1 H, d, ^3^*J*_H,H_ = 9.36 Hz, Ar-H), 7.46 (1 H, d, ^3^*J*_H,H_ = 9.18 Hz, Ar-H), 8.41 (2 H, br, NH_2_). ^13^C-NMR (150 MHz; CD_2_Cl_2_) δ = 55.61, 55.71, 56.67, 97.63, 97.70, 99.01, 99.87, 105.42, 112.51, 115.80, 117.61, 119.99, 131.52, 131.77, 133.60, 156.76, 158.05, 159.89, 162.16, 162.27, 163.45, 167.36. IR (ATR): v = 3070 (m) cm^−1^ (NH_2_^+^), 1693 (m) cm^−1^ (NH_2_^+^). HR MS (ES) m/z = found 362.1387, calculated 362.1392 [M]^+^.

### 6-Methoxy-9-(1-naphthyl)xanthen-3-iminium trifluoroacetate (22)

1-bromonaphthalene (1.80 g, 8.70 mmol, 1.22 ml), magnesium turnings (0.22 g, 9.06 mmol) × 3 portions. 2-chlorotritylchloride resin bound 3-methoxy-6-(tritylamino)xanthen-9-one (0.21 mg/mg loading by qNMR, 0.84 g resin, 0.18 g xanthone, 0.725 mmol). Purification achieved *via* flash column chromatography using gradient elution 2–10% methanol:DCM to yield a foamy red solid. (0.45 g, 13.3%, 1:99 methanol:DCM R_f_ = 0.1). ^1^H-NMR (600 MHz; CD_2_Cl_2_) δ = 4.02 (3 H, s, CH_3_), 6.92 (1 H, dd, ^4^*J*_H,H_ = 2.46, ^3^*J*_H,H_ = 9.18 Hz, Ar-H), 6.98 (1 H, dd, ^4^*J*_H,H_ = 2.07, ^3^*J*_H,H_ = 9.42 Hz, Ar-H), 7.10 (1 H, d, ^4^*J*_H,H_ = 2.07 Hz, Ar-H), 7.17 (1 H, d, ^3^*J*_H,H_ = 9.42 Hz, Ar-H), 7.18 (1 H, d, ^3^*J*_H,H_ = 9.18 Hz, Ar-H), 7.26–7.27 (2 H, m, Ar-H), 7.40 (1 H, ddd, ^4^*J*_H,H_ = 1.34, ^3^*J*_H,H_ = 6.93, ^3^*J*_H,H_ = 8.36 Hz, Ar-H), 7.47 (1 H, dd, ^4^*J*_H,H_ = 1.05, ^3^*J*_H,H_ = 7.01 Hz, Ar-H), 7.58 (1 H, ddd, ^4^*J*_H,H_ = 1.14, ^3^*J*_H,H_ = 6.93, ^3^*J*_H,H_ = 8.34 Hz, Ar-H), 7.71 (1 H, dd, ^3^*J*_H,H_ = 8.31, ^3^*J*_H,H_ = 7.01 Hz, Ar-H), 8.04 (1 H, d, ^3^*J*_H,H_ = 8.34 Hz, Ar-H), 8.16 (1 H, d, ^3^*J*_H,H_ = 8.31 Hz, Ar-H), 8.77 (1 H, br, NH_2_). ^13^C-NMR (150 MHz; CD_2_Cl_2_) δ = 57.18, 98.46, 100.57, 116.34, 117.16, 118.53, 121.16, 125.36, 125.51, 127.39, 128.02, 128.19, 129.12, 129.45, 131.19, 131.69, 131.77, 133.61, 133.86, 157.10, 158.81, 160.16, 163.10, 168.08. IR (ATR): v = 3064 (m) cm^−1^ (NH_2_^+^), 1674 (m) cm^−1^ (NH_2_^+^). HR MS (ES) m/z = found 352.1353, requires 352.1338 [M]^+^.

### 6-Methoxy-9-[2-(trifluoromethyl)phenyl]xanthen-3-iminium trifluoroacetate (23)

Under a dry nitrogen atmosphere, Amberlyst A21 basic resin (0.50 g) was washed with a 5:1 DCM/methanol mixture (2 × 10 ml), followed by DCM (5 × 10 ml), methanol (5 × 10 ml) and an addition DCM wash (5 × 10 ml). The resin was then dried under a stream of nitrogen followed by high vacuum. SG2.HCl (0.01 g, 0.0296 mmol) was then reconstituted into DCM/methanol solution of 5:1 (20 ml), to which the clean dry Amberlyst A21 resin was then added and stirred for 20 h. The resin was filtered off and washed several times with the reaction solution. The washings were combined, the solvent removed under highvacuum and stored under nitrogen. The dry compound was reconstituted in a 10% trifluoroacetic acid solution in DCM and stirred under nitrogen for 24 h, the solvent was then removed under high vacuum for several days, to yield a deep red solid. (0.012 g, 0.0248 mmol, 84%, 1:99 methanol:DCM R_f_ = 0.1). ^1^H-NMR (600 MHz; MeOD) δ = 4.08 (3 H, s, CH_3_), 6.99 (1 H, d, ^4^*J*_H,H_ = 2.04 Hz, Ar-H), 7.05 (1 H, dd, ^4^*J*_H,H_ = 2.04, ^3^*J*_H,H_ = 9.42 Hz, Ar-H), 7.16 (1 H, dd, ^4^*J*_H,H_ = 2.40, ^3^*J*_H,H_ = 9.42 Hz, Ar-H), 7.23 (1 H, d, ^3^*J*_H,H_ = 9.42 Hz, Ar-H), 7.24 (1 H, d, ^3^*J*_H,H_ = 9.42 Hz, Ar-H), 7.47 (1 H, d, ^4^*J*_H,H_ = 2.40 Hz, Ar-H), 7.54–7.55 (1 H, m, Ar-H), 7.91–7.93 (2 H, m, Ar-H), 8.06–8.08 (1 H, m, Ar-H). ^13^C-NMR (150 MHz; MeOD) δ = 57.60, 98.55, 101.42, 117.02, 118.26, 118.81, 121.48, 128.15, 130.01, 130.22, 131.27, 132.03, 132.19, 132.35, 133.91, 134.51, 158.14, 158.21, 161.31, 164.46, 169.72. ^19^F-NMR (376 MHz; CD_2_Cl_2_) δ = −59.17, −76.23. IR (ATR): v = 3410 (s) cm^−1^ (NH_2_^+^), 1645 (m) cm^−1^ (NH_2_^+^). HR MS (ES) m/z = found 370.1060, requires 370.1055 [M]^+^.

### [3-Acetamido-6-methoxy-9-(o-tolyl)xanthen-9-yl] acetate (24)

Into a stirred solution of (**15**) (0.01 g, 0.0230 mmol) in anhydrous pyridine under a dry nitrogen atmosphere, acetic anhydride (0.01 g, 0.115 mmol, 0.01 ml) was added dropwise. The resulting solution was stirred at room temperature for 16 h while monitoring *via* TLC analysis, and then evaporated to dryness under high vacuum at 40 °C. The crude mixture was reconstituted in DCM (10 ml), washed with 0.5 M aqueous HCl solution (3 × 5 ml), saturated NaHCO_3_ (2 × 5 ml), brine (1 × 10 ml), dried over anhydrous Na_2_SO_4_ and finally evaporated to dryness under high vacuum. Purification was achieved *via* flash column chromatography using a gradient elution of 0–5% methanol:DCM to yield an orange solid. (0.007 g, 3%, 1:9 methanol:DCM R_f_ = 0.4). ^1^H-NMR (600 MHz; CD_2_Cl_2_) δ = 1.43 (3 H, s, CH_3_), 2.14 (3 H, s, CH_3_), 2.73 (3 H, s, CH_3_), 3.82 (3 H, s, CH_3_), 6.59 (1 H, dd, ^4^*J*_H,H_ = 2.52, ^3^*J*_H,H_ = 8.70 Hz, Ar-H), 6.67 (1 H, d, ^4^*J*_H,H_ = 2.52 Hz, Ar-H), 6.85 (1 H, d, ^3^*J*_H,H_ = 8.70 Hz, Ar-H), 6.89 (1 H, d, ^3^*J*_H,H_ = 8.46 Hz, Ar-H), 6.92 (1 H, dd, ^4^*J*_H,H_ = 1.95, ^3^*J*_H,H_ = 8.46 Hz, Ar-H), 6.95 (1 H, dd, ^3^*J*_H,H_ = 7.41, ^4^*J*_H,H_ = 0.78 Hz, Ar-H), 7.18 (1 H, ddd, ^3^*J*_H,H_ = 7.41, ^3^*J*_H,H_ = 7.41, ^4^*J*_H,H_ = 1.38 Hz, Ar-H), 7.26 (1 H, br, NH), 7.33 (1 H, ddd, ^3^*J*_H,H_ = 7.92, ^3^*J*_H,H_ = 7.41, ^4^*J*_H,H_ = 0.78 Hz, Ar-H), 7.63 (1 H, d, ^4^*J*_H,H_ = 1.95 Hz, Ar-H), 8.28 (1 H, dd, ^4^*J*_H,H_ = 1.38, 7.92 Hz, Ar-H). ^13^C-NMR HSQC (150 MHz; CD_2_Cl_2_) δ = 131.76, 130.03, 129.60, 127.32, 125.10, 114.58, 114.58, 111.08, 99.50, 55.40, 49.18, 24.52, 20.08. IR (ATR): v = 3301 (m) cm^−1^ (NH). HR MS (ES) m/z = found 358.1442, requires 358.1443 [M − OAc]^+^.

### N-[6-methoxy-9-(o-tolyl)xanthen-3-ylidene]acetamide (25)

[3-Acetamido-6-methoxy-9-(o-tolyl)xanthen-9-yl] acetate was exposed to sunlight over 70 h at 20 °C to induce photolysis to form the elimination product SG1Ac as an orange brown solid. (0.006 g, 100%). ^1^H-NMR (600 MHz; CD_2_Cl_2_) δ = 2.46 (3 H, s, CH_3_), 3.41 (3 H, s, CH_3_), 4.15 (3 H, s, CH_3_), 7.23 (1 H, dd, ^4^*J*_H,H_ = 1.26, ^3^*J*_H,H_ = 7.56 Hz, Ar-H), 7.25 (1 H, dd, ^4^*J*_H,H_ = 2.34, 9.24 Hz, Ar-H), 7.44 (1 H, d, ^4^*J*_H,H_ = 2.34 Hz, Ar-H), 7.49 (1 H, ddd, ^4^*J*_H,H_ = 0.78, ^3^*J*_H,H_ = 7.56, ^3^*J*_H,H_ = 7.65 Hz, Ar-H), 7.52 (1 H, dd, ^3^*J*_H,H_ = 7.65, ^4^*J*_H,H_ = 0.78 Hz, Ar-H), 7.58 (1 H, d, ^3^*J*_H,H_ = 9.24 Hz, Ar-H), 7.59 (1 H, d, ^3^*J*_H,H_ = 9.42 Hz, Ar-H), 7.62 (1 H, ddd, ^4^*J*_H,H_ = 1.26, ^3^*J*_H,H_ = 7.65, ^3^*J*_H,H_ = 7.65 Hz, Ar-H), 8.56 (1 H, d, ^3^*J*_H,H_ = 9.42 Hz, Ar-H), 9.02 (1 H, d, ^4^*J*_H,H_ = 1.10 Hz, Ar-H). ^13^C-NMR HSQC (150 MHz; CD_2_Cl_2_) δ = 131.89, 131.23, 131.10, 128.80, 126.30, 120.35, 99.89, 57.66, 25.13, 19.67. IR (ATR): v = 2922 (m) cm^−1^ (Ar-CH), 1706 (m) cm^−1^ (C=N). HR MS (ES) m/z = found 358.1446, requires 358.1443 [M]^+^.

### [3-Acetamido-6-methoxyl-9-(2-methoxy-5-methyl-phenyl) xanthen-9-yl] acetate (26)

Into a stirred solution of SG5.TFA (0.042 g, 0.0910 mmol) in anhydrous pyridine under a dry nitrogen atmosphere, acetic anhydride (0.047 g, 0.457 mmol, 0.044 ml) was added dropwise. The resulting solution was stirred at room temperature for 16 h while monitoring *via* TLC analysis, after which an additional portion of acetic anhydride (0.141 g, 1.37 mmol, 0.132 ml) was added dropwise. After an additional 4 h the reaction mixture was evaporated to dryness under high vacuum at 40 ^o^C. The crude mixture was reconstituted in DCM (10 ml), washed with 0.5 M aqueous HCl solution (3 × 5 ml), saturated NaHCO_3_ (2 × 5 ml), brine (1 × 10 ml), dried over anhydrous Na_2_SO_4_ and finally evaporated to dryness under high vacuum. Purification was achieved *via* flash column chromatography using a gradient elution of 0–5% methanol:DCM to yield an deep orange solid. (0.010 g, 25%, 1:9 methanol:DCM R_f_ = 0.4). ^1^H-NMR (600 MHz; CD_2_Cl_2_) δ = 2.13 (3 H, s, CH_3_), 2.39 (3 H, s, CH_3_), 2.79 (3 H, s, CH_3_), 3.11 (3 H, s, CH_3_), 3.80 (3 H, s, CH_3_), 6.55 (1 H, dd, ^4^*J*_H,H_ = 2.55, ^3^_H,H_ = 8.58 Hz, Ar-H), 6.57 (1 H, d, ^3^*J*_H,H_ = 8.22 Hz, Ar-H), 6.65 (1 H, d, ^4^*J*_H,H_ = 2.55 Hz, Ar-H), 6.91 (1 H, dd, ^4^*J*_H,H_ = 1.89, ^3^*J*_H,H_ = 8.46 Hz, Ar-H), 6.95 (1 H, d, ^3^*J*_H,H_ = 8.58 Hz, Ar-H), 6.99 (1 H, d, ^3^*J*_H,H_ = 8.46 Hz, Ar-H), 7.02 (1 H, dd, ^4^*J*_H,H_ = 2.28, ^3^*J*_H,H_ = 8.22 Hz, Ar-H), 7.30 (1 H, s, NH), 7.54 (1 H, d, ^4^*J*_H,H_ = 1.89 Hz, Ar-H), 7.93 (1 H, d, ^4^*J*_H,H_ = 2.28 Hz, Ar-H). ^13^C-NMR (150 MHz; CD_2_Cl_2_) δ = 21.08, 24.83, 49.98, 55.75, 56.30, 73.10, 99.98, 106.36, 110.38, 113.53, 114.34, 115.71, 119.44, 126.17, 128.89, 129.01, 129.33, 129.87, 137.42, 138.68, 152.91, 153.45, 154.29, 160.27, 168.56. IR (ATR): v = 3300 (w) cm^−1^ (NH). HR MS (ES) m/z = found 388.1553, requires 388.1549 [M − OAc]^+^.

### [3-Acetamido-6-methoxy-9-(naphthyl)xanthen-9-yl] acetate (28)

Into a stirred solution of SG8.TFA (0.079 g, 0.170 mmol) in anhydrous pyridine under a dry nitrogen atmosphere, acetic anhydride (0.868 g, 8.50 mmol, 0.803 ml) was added dropwise. The resulting solution was stirred at room temperature for 14 h while monitoring *via* TLC analysis, after which it was evaporated to dryness under high vacuum at 40 ^o^C. The crude mixture was reconstituted in DCM (10 ml), washed with 0.5 M aqueous HCl solution (3 × 5 ml), saturated NaHCO_3_ (2 × 5 ml), brine (1 × 10 ml), dried over anhydrous Na_2_SO_4_ and finally evaporated to dryness under high vacuum. Purification was achieved *via* flash column chromatography using a gradient elution of 0–5% methanol:DCM to yield a deep orange solid. (0.014 g, 18%, 1:9 methanol:DCM R_f_ = 0.4). ^1^H-NMR (600 MHz; CD_2_Cl_2_) δ = 2.12 (3 H, s, CH_3_), 2.80 (3 H, s, CH_3_), 3.80 (3 H, s, CH_3_), 6.50 (1 H, dd, ^4^*J*_H,H_ = 2.55, ^3^*J*_H,H_ = 8.70 Hz, Ar-H), 6.77 (1 H, d, ^4^*J*_H,H_ = 2.55 Hz, Ar-H), 6.79 (1 H, dd, ^4^*J*_H,H_ = 2.04, ^3^*J*_H,H_ = 8.46 Hz, Ar-H), 6.82 (1 H, d, ^3^*J*_H,H_ = 8.70 Hz, Ar-H), 6.84 (1 H, d, ^3^*J*_H,H_ = 8.46 Hz, Ar-H), 7.00 (1 H, ddd, ^4^*J*_H,H_ = 1.55, ^3^*J*_H,H_ = 6.84, ^3^*J*_H,H_ = 8.69 Hz, Ar-H), 7.20–7.24 (1 H, m, Ar-H), 7.23 (1 H, ddd, ^4^*J*_H,H_ = 1.11, ^3^*J*_H,H_ = 6.84 Hz, ^3^*J*_H,H_ = 8.04, Ar-H), 7.32 (1 H, s, NH), 7.62 (1 H, dd, ^3^*J*_H,H_ = 7.41, ^3^*J*_H,H_ = 8.10 Hz, Ar-H), 7.76–7.78 (2 H, m, Ar-H), 7.84 (1 H, d, ^3^*J*_H,H_ = 8.10 Hz, Ar-H), 8.52 (1 H, dd, ^4^*J*_H,H_ = 0.69, ^3^*J*_H,H_ = 7.41 Hz, Ar-H). ^13^C-NMR (150 MHz; CD_2_Cl_2_) δ = 24.83, 49.51, 55.82, 74.65, 100.33, 106.69, 111.41, 115.06, 115.64, 119.16, 125.00, 125.02, 125.05, 125.18, 125.63, 129.13, 129.25, 129.82, 130.24, 130.27, 135.04, 139.42, 141.44, 152.48, 153.06, 160.89, 168.66, 168.68. IR (ATR): v = 3306 (m) cm^−1^ (NH). HR MS (ES) m/z = found 394.1444, requires 394.1443 [M + H − OAc]^+^.

## Conclusions

In addition to the synthesis and characterisation of novel pro-fluorescent Singapore Greens analogues we report favourable fluorescence and biophysical properties. The favourability of the fluorescence profile and the ease of functionalisation further evidences the concept of future probe generation based upon the SG core. These novel derivatives further prove that the upper carboxyl group is not essential for fluorescence, while upper ring electron density modulates QY though a PeT-based quenching mechanism. Of considerable interest is the observation that the correlation between electron density and QY is not linear, rather a threshold exists beyond which PeT predominates and quantum yield drops significantly. This correlates structure with activity and allows prediction of the properties of future analogues without the need for synthesis, as well as allowing rationale design of analogues with tailored properties. Functionalisation *via* acylation of the NH_2_ group of analogue (**15**) produced (**25**), a prospective proof of concept protease probe. This was subsequently treated with several protease enzymes under physiological conditions, proteolytic cleavage was observed to occur with notable selectivity, releasing (**15**) to elicit a large increase in fluorescence as expected. This observed proteolysis provided proof of concept that Singapore Green (**15**), and by extension its analogues may be utilised as pro-fluorescent cores for future novel and selective protease probes.

## Supplementary material

Supplementary material.
