# Supplementary material for: Synthesis and Spectral Properties of Novel Singapore Green Analogues for Protease Detection
Source: Sci Rep. 2020 Jan 14;10:259. doi: 10.1038/s41598-019-57124-0 (PMC6959322; doi:10.1038/s41598-019-57124-0)
Supplement: Supplementary file 1 — Supplementary material. [file 41598_2019_57124_MOESM1_ESM.docx]

**Supplementary Information**

**ppm**

**25**

**24**

**Figure S1.** Time lapse ^1^H-NMR of the photolysis of (**24**) into (**25**) (aliphatic region).

**Excitation vs Emission Maxima for Singapore Greens**


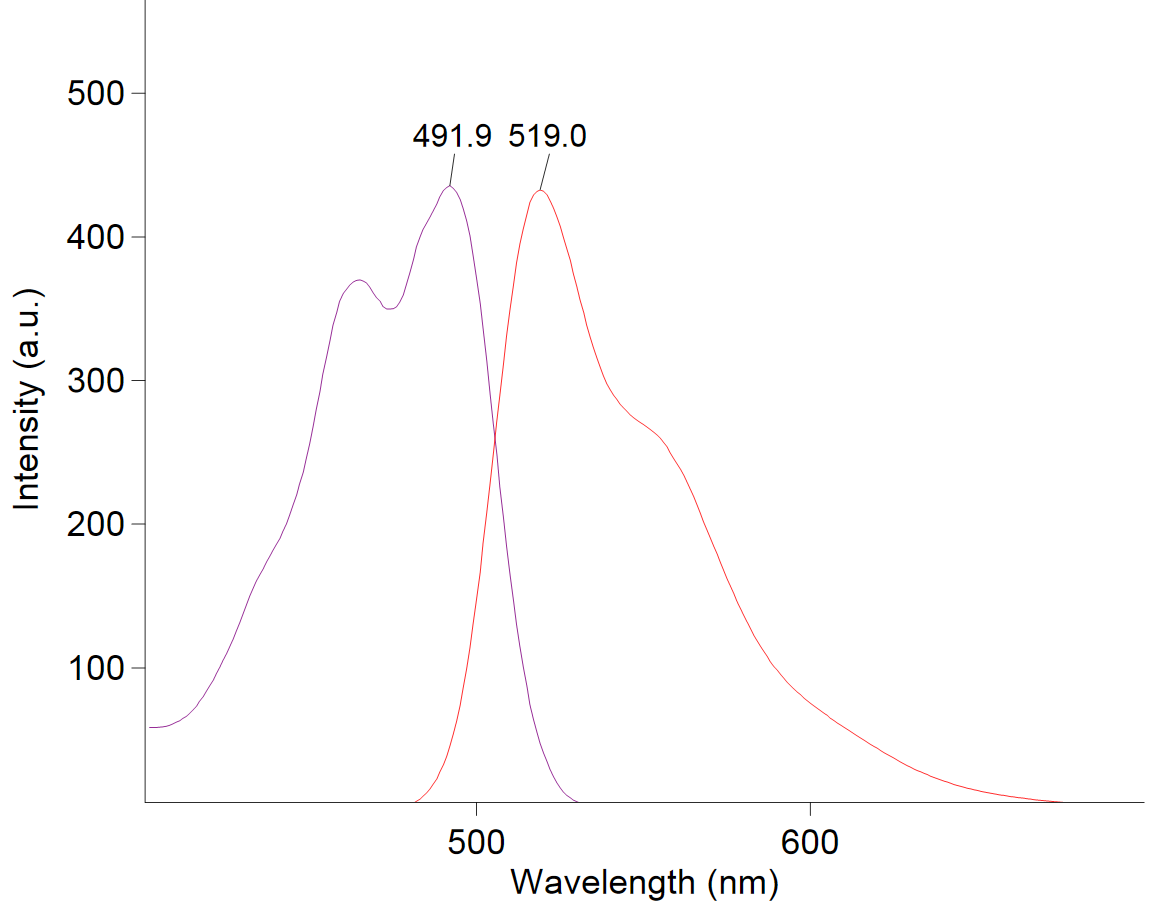


**Figure S2.** Compound (15) in absolute EtOH.


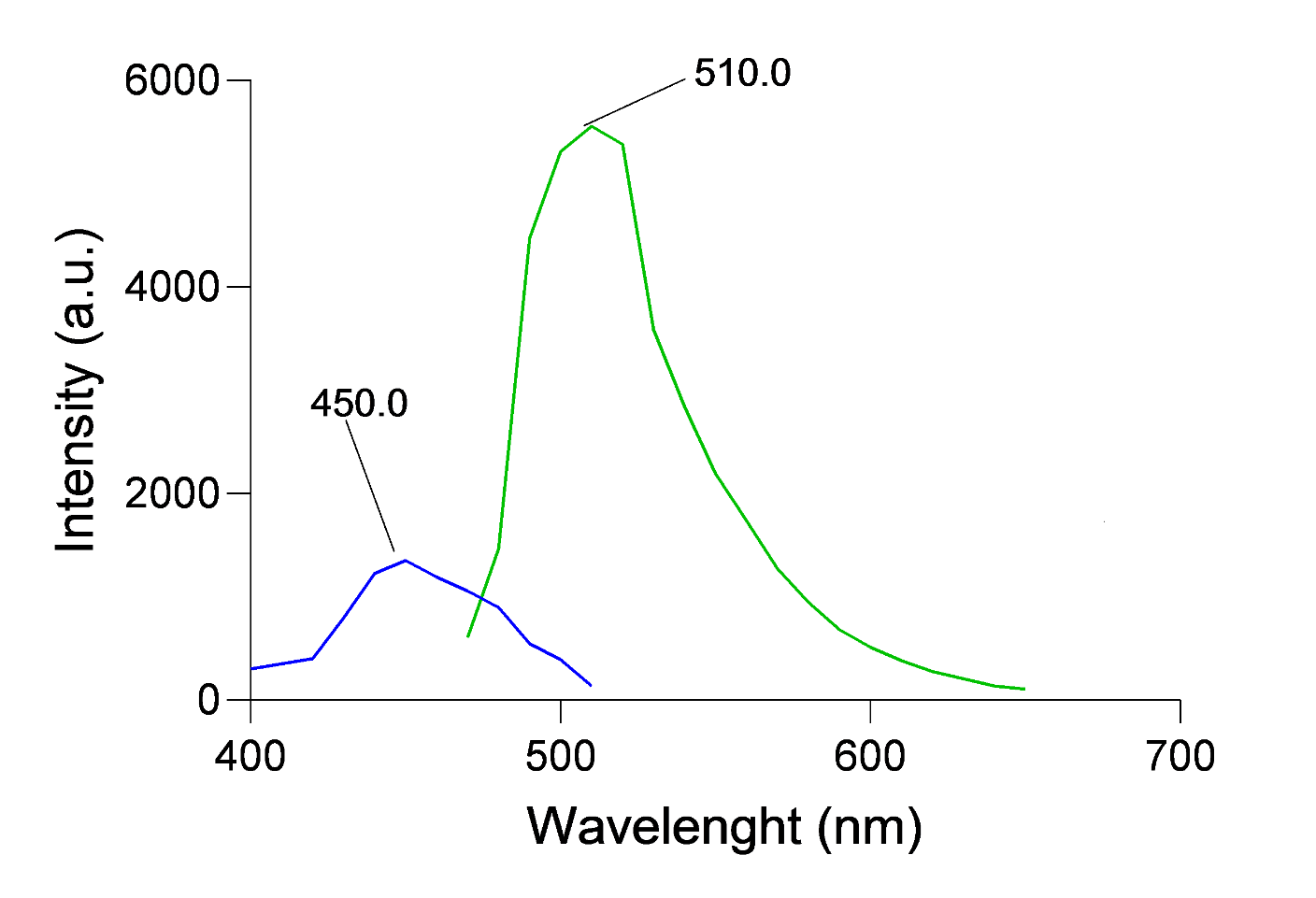
.

Intensity (a.u.)

Wavelength (nm)

**Figure S3**. Compound (**15**) in PBS + 5 % EtOH

.


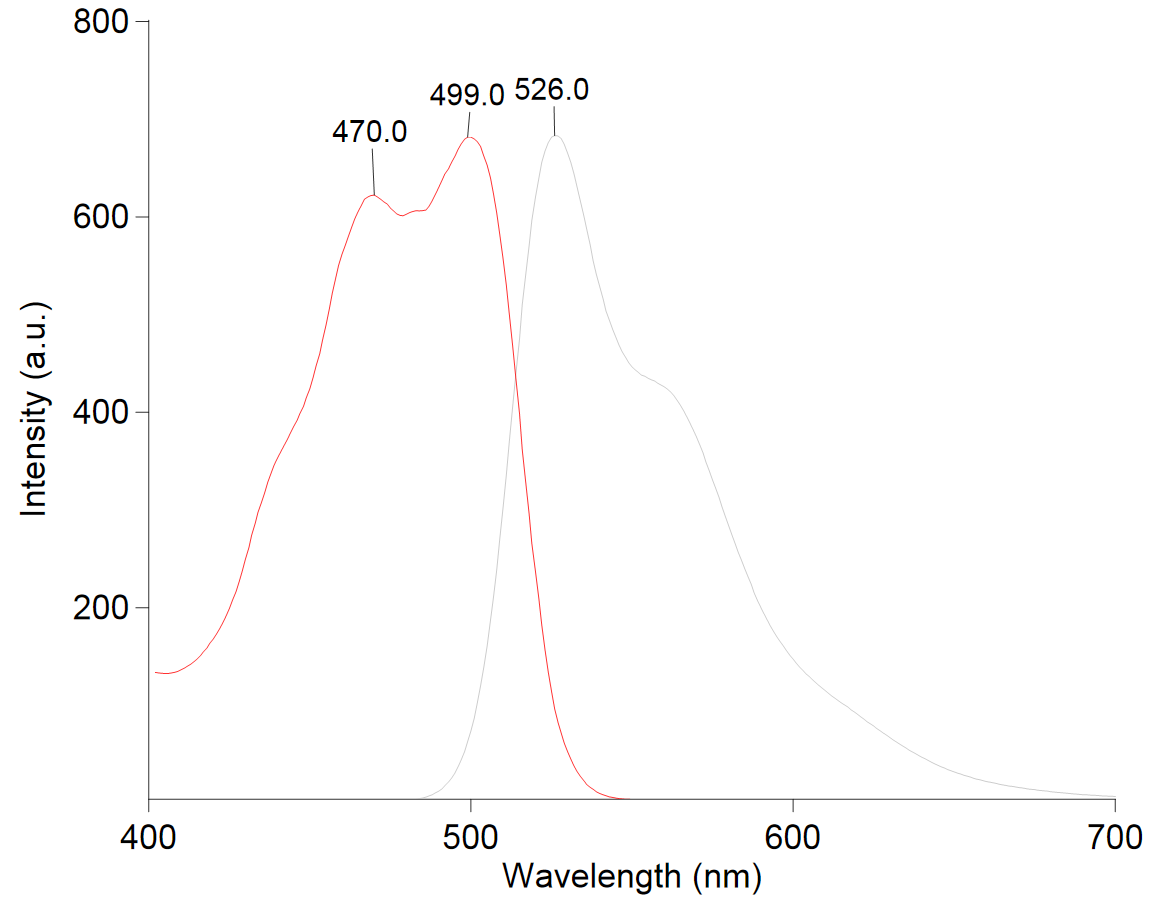


**Figure S4.** Compound (**16**) in absolute EtOH


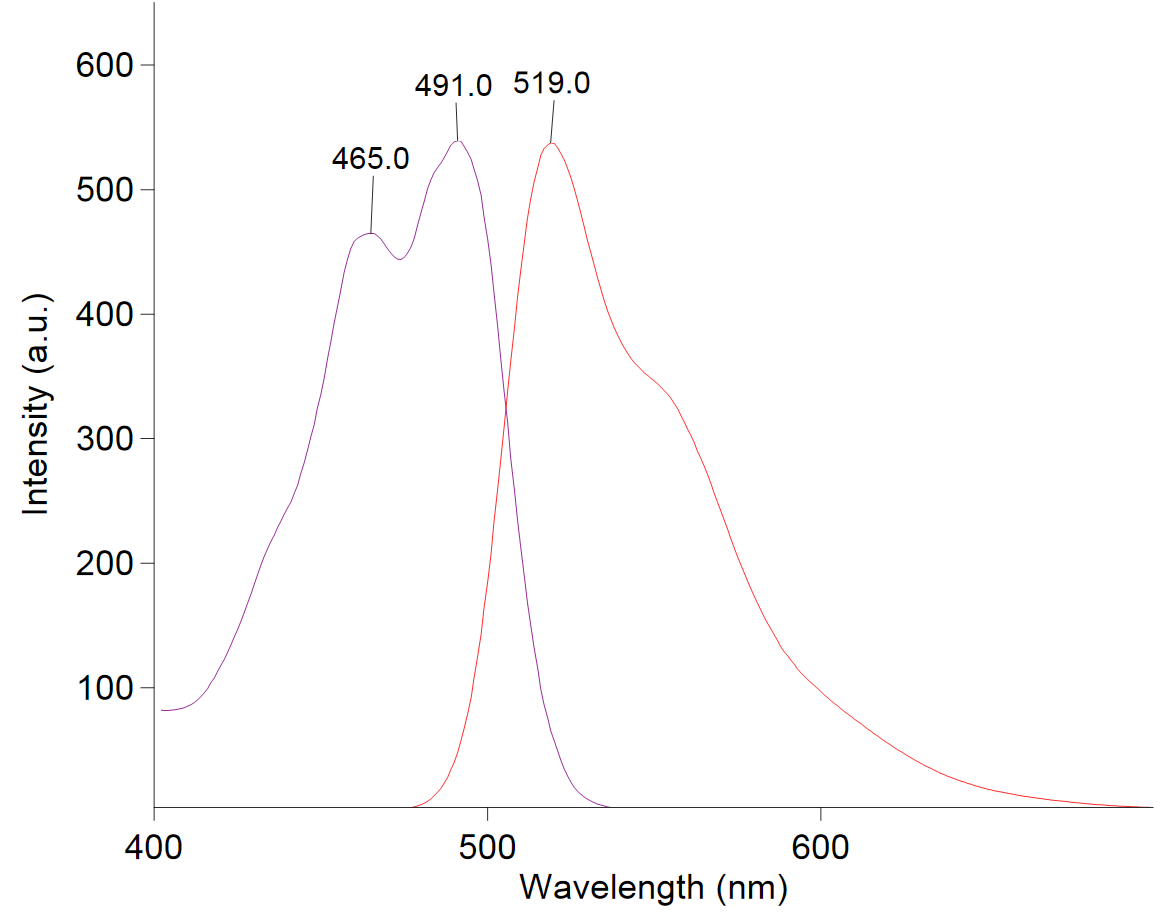


**Figure S5.** Compound (**17**) in absolute EtOH.


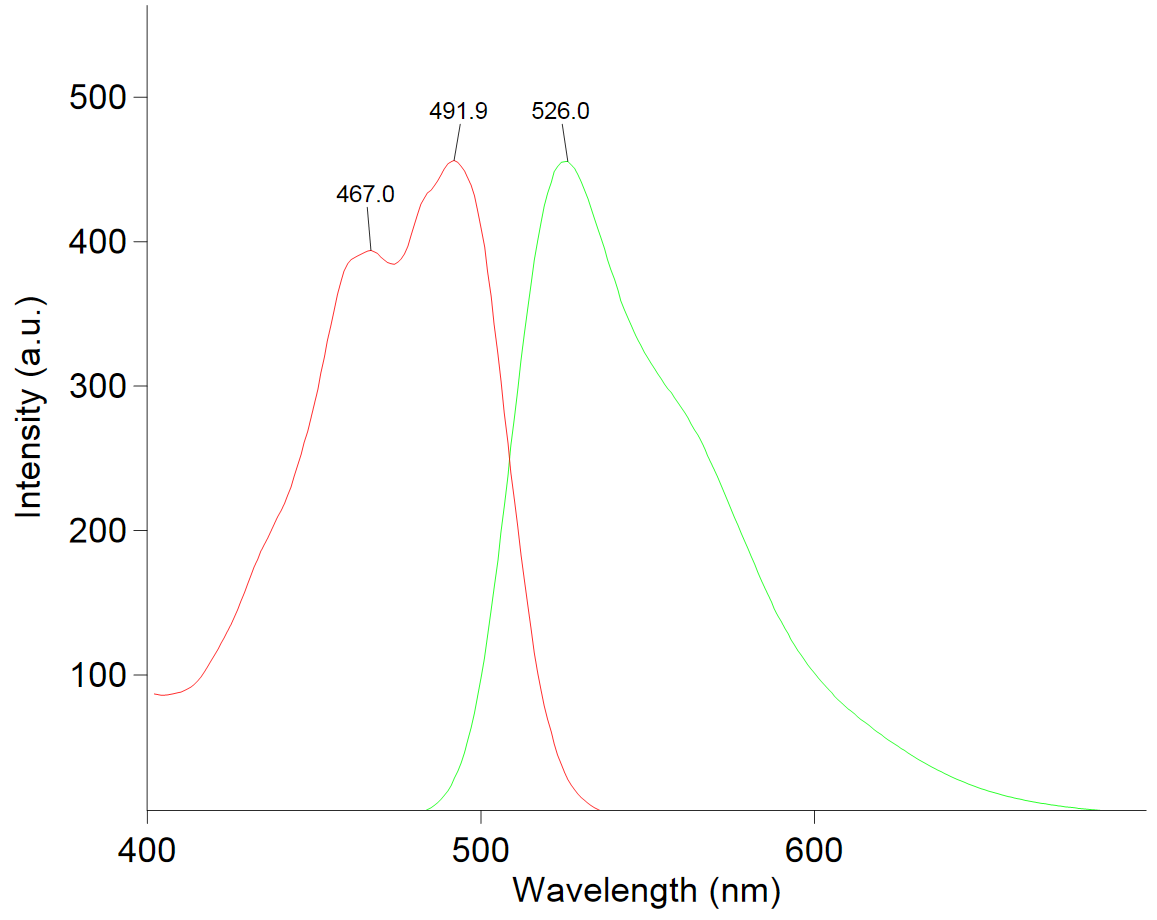


**Figure S6.** Compound (**18**) in absolute EtOH.


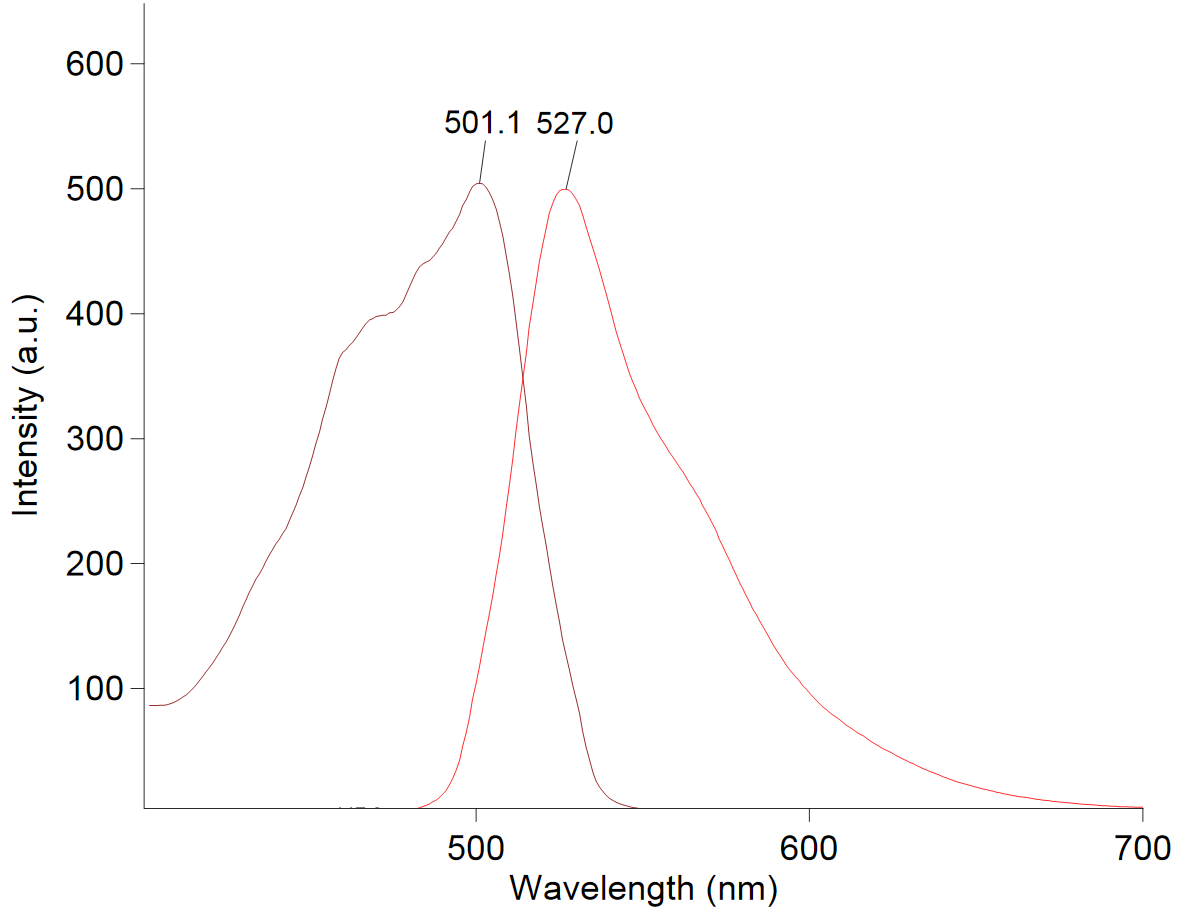


**Figure S7.** Compound (**19**) in absolute EtOH.


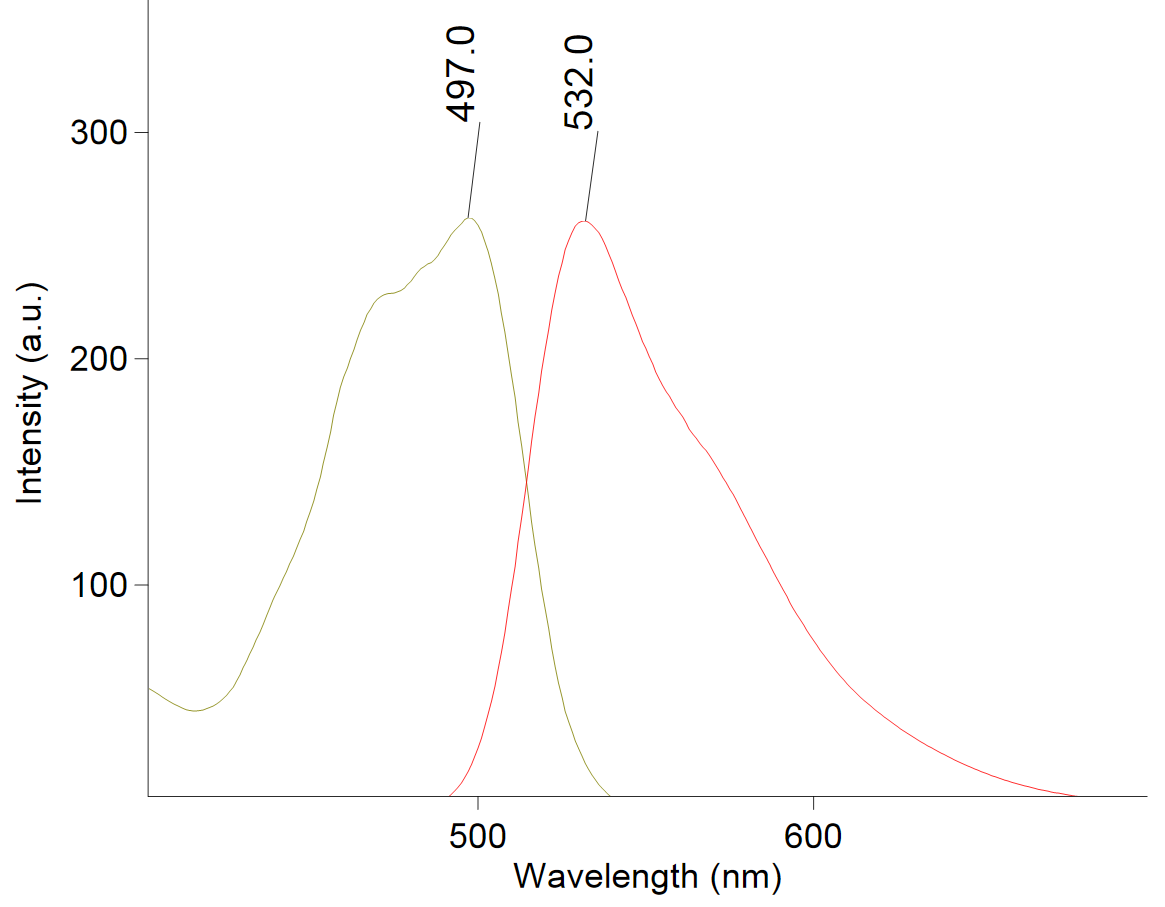


**Figure S8.** Compound (**20**) in absolute EtOH.


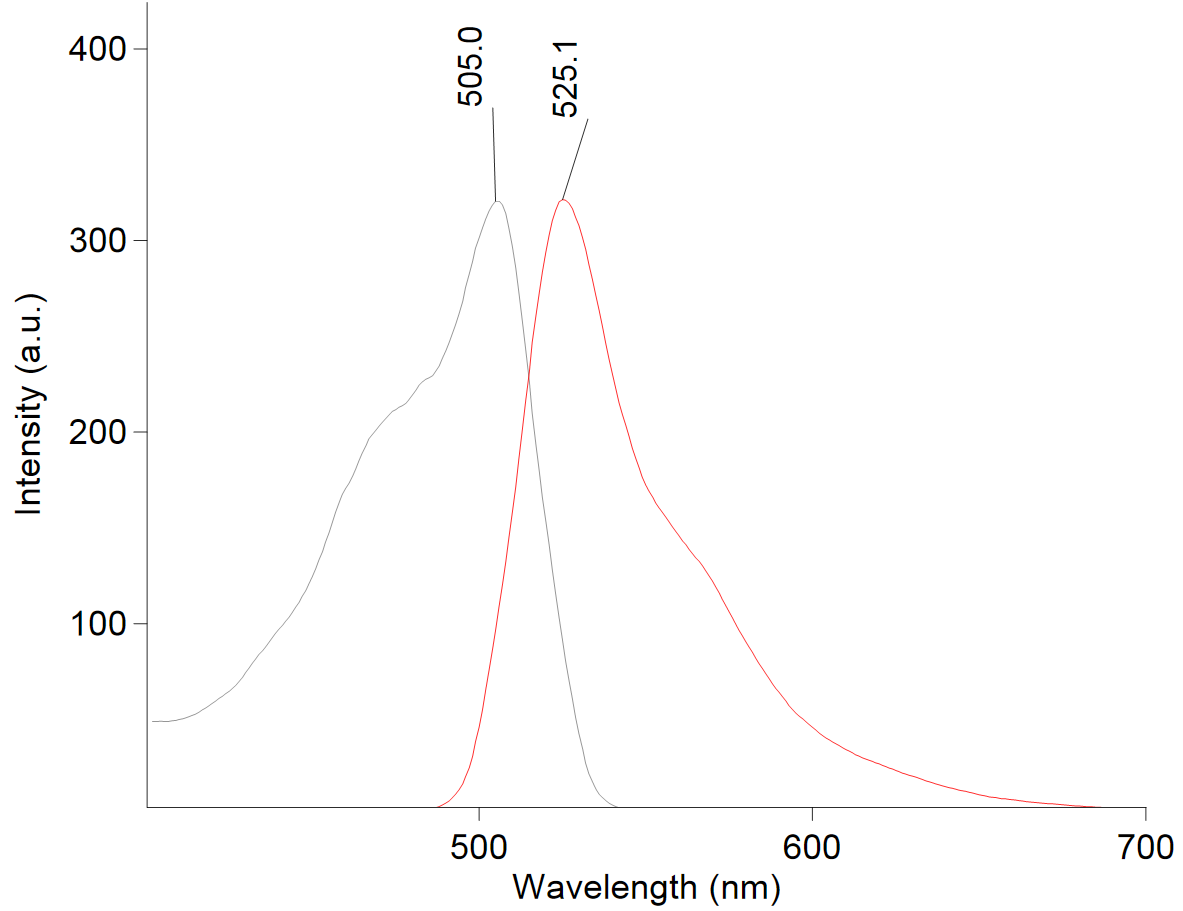


**Figure S9.** Compound (**21**) in absolute EtOH.


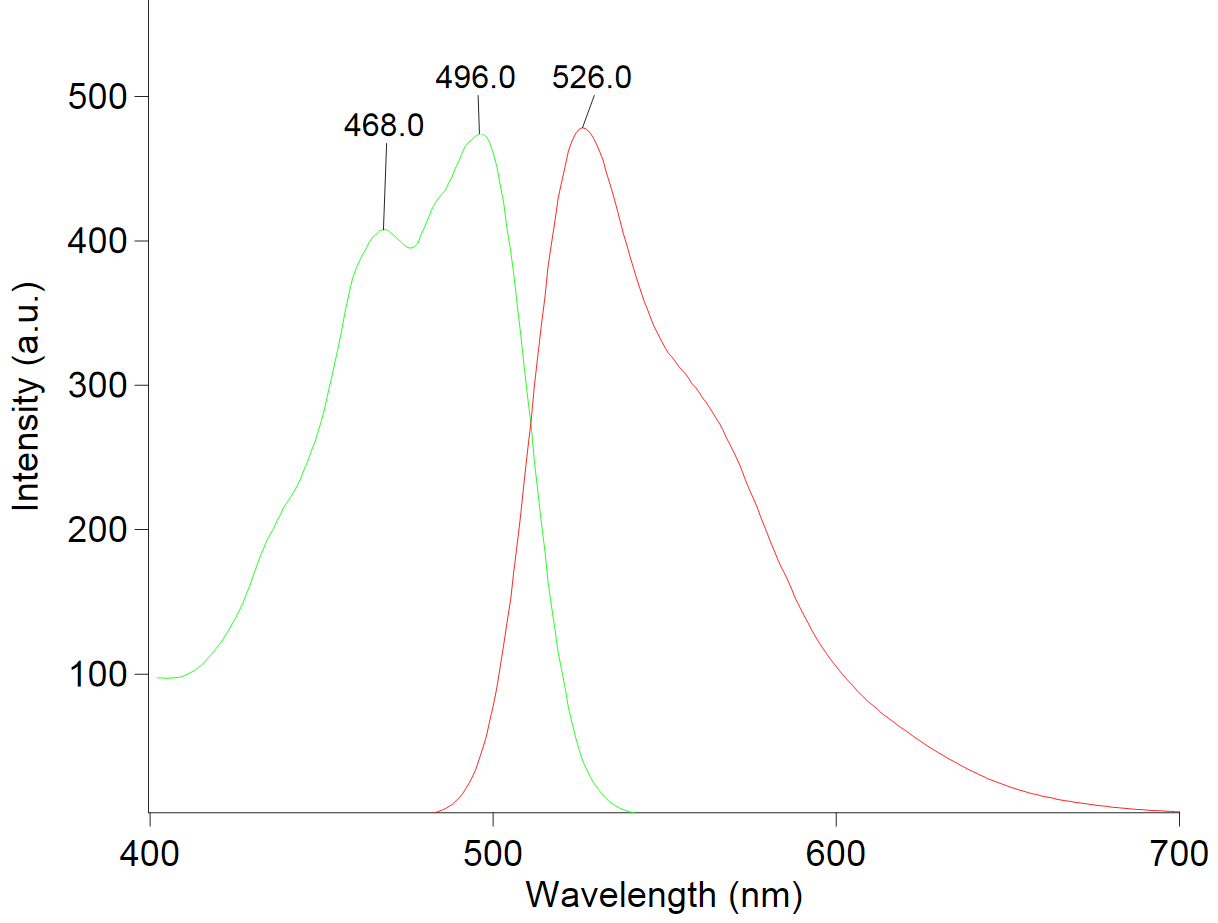


**Figure S10.** Compound (**22**) in absolute EtOH.


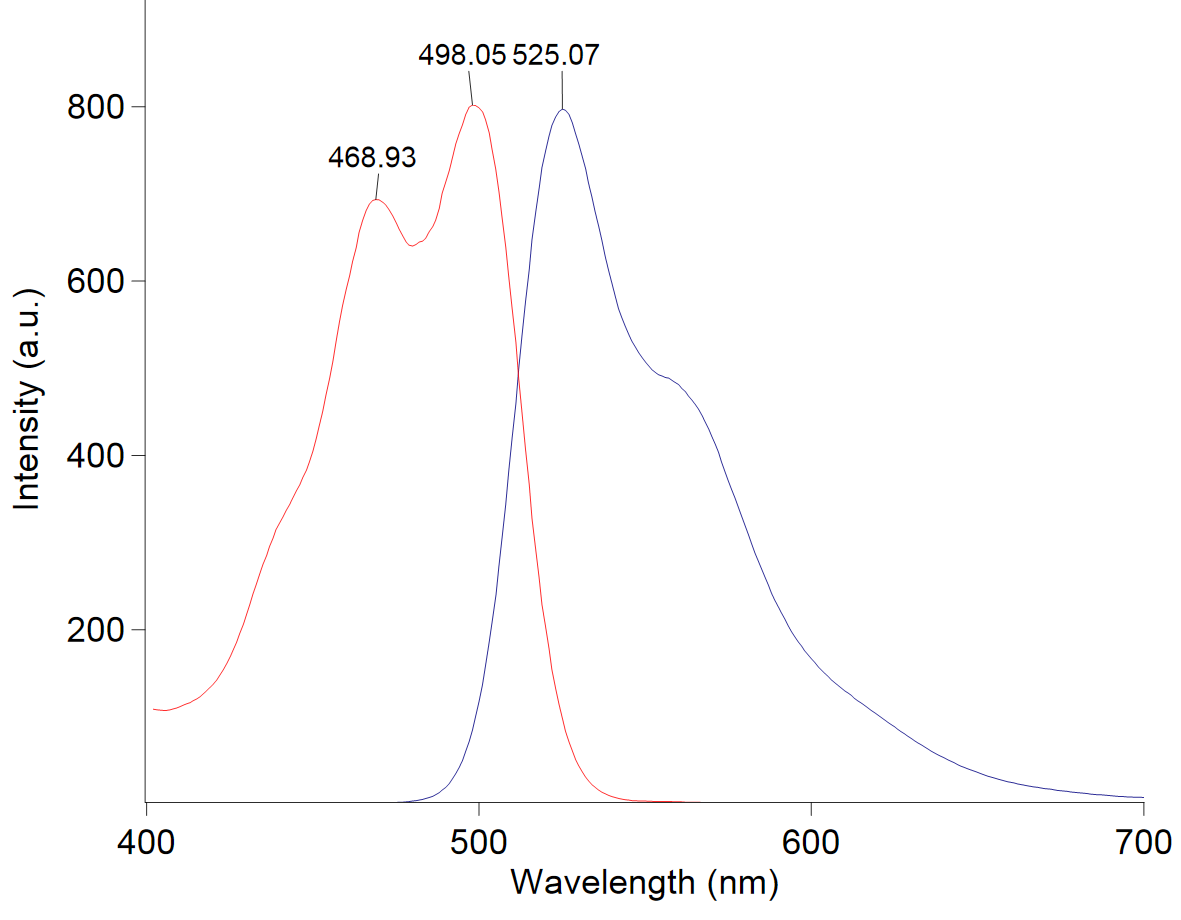


**Figure S11**. Compound (**23**) in absolute EtOH.


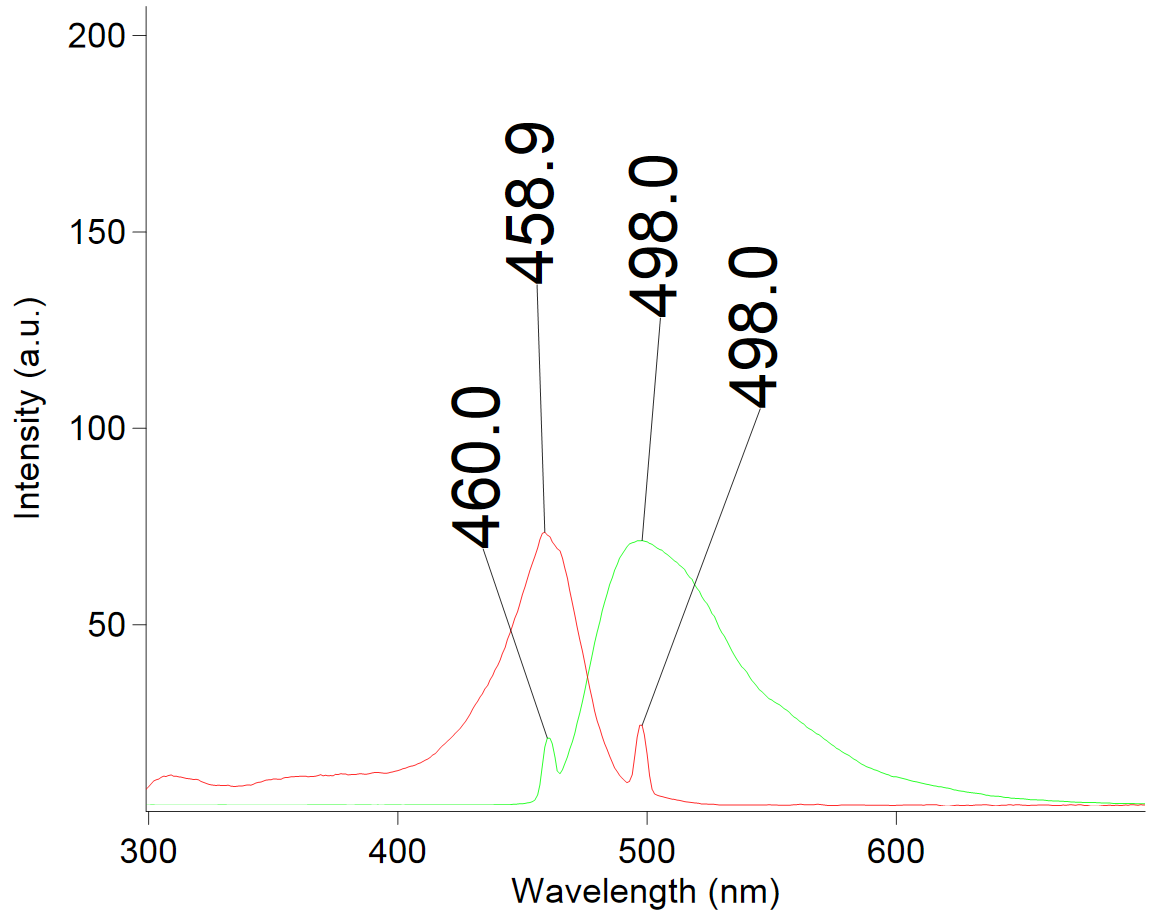


**Figure S12.** Compound (**24**) in absolute EtOH.


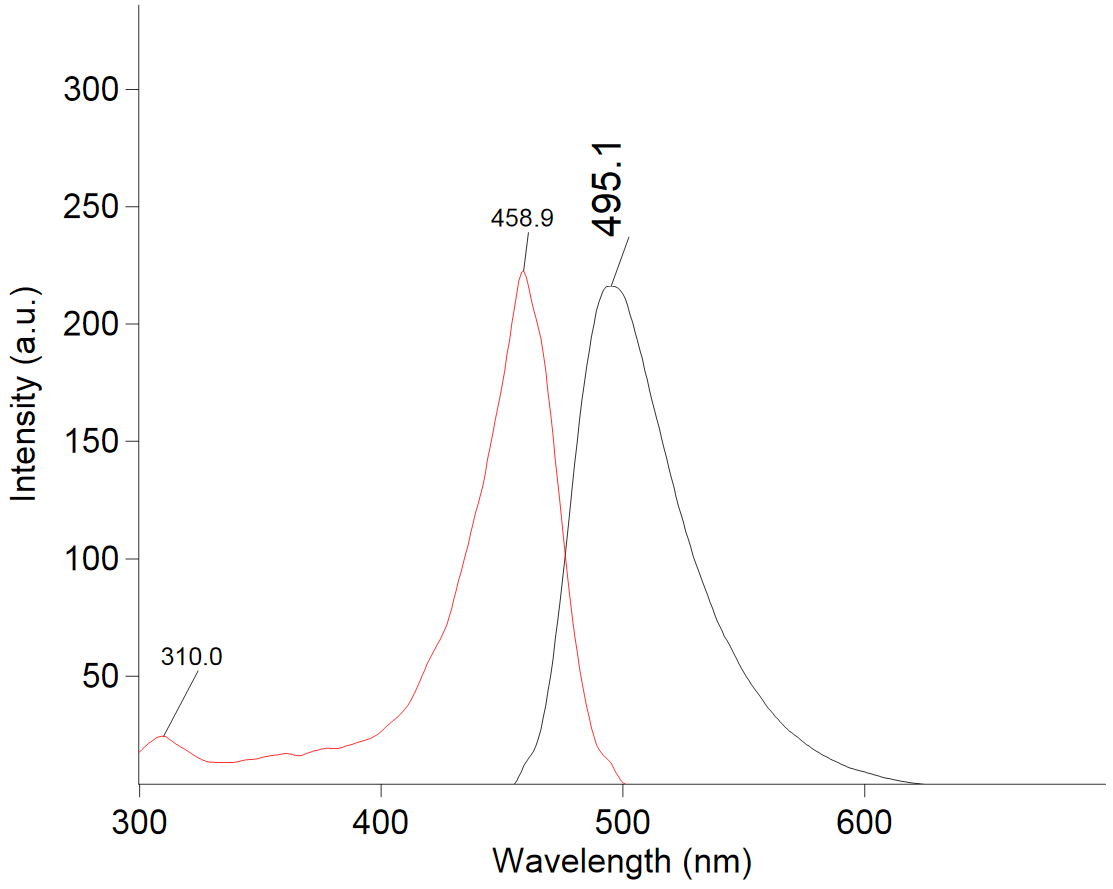


**Figure S13.** Compound (**25**) in absolute EtOH.

**
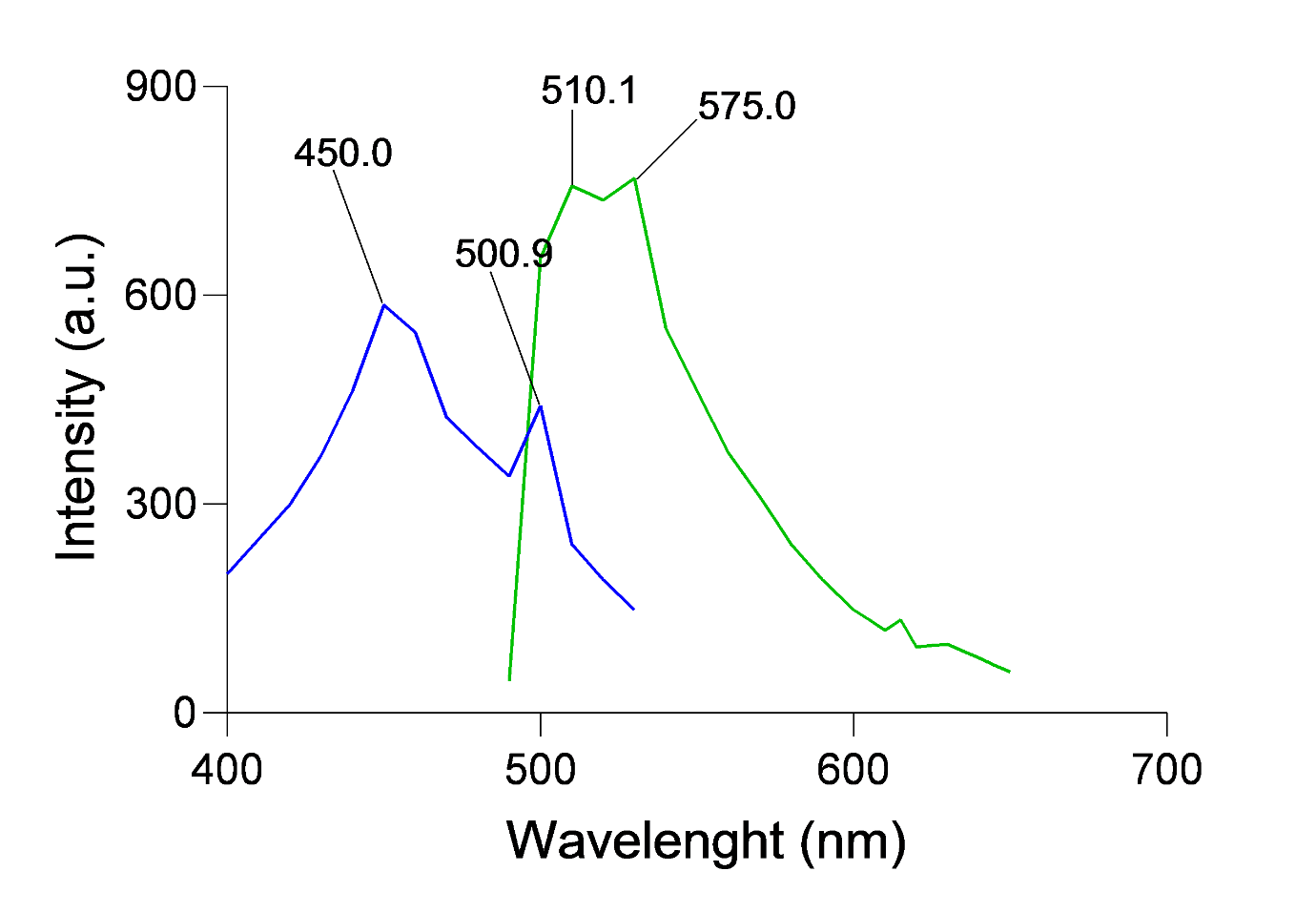
**.

Intensity (a.u.)

Wavelength (nm)

**Figure S14.** Compound (**25**) in PBS + 5 % EtOH


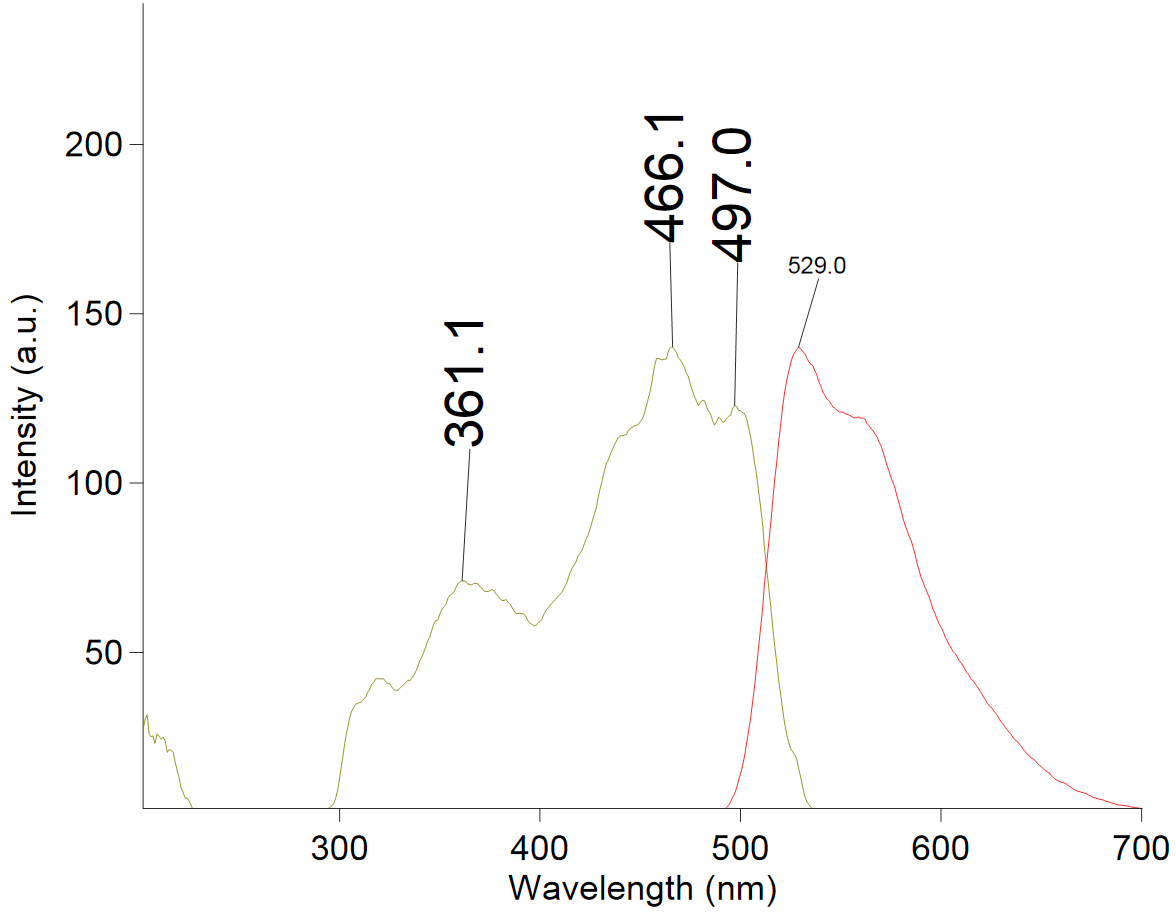


**Figure S15.** Compound (**26**) in absolute EtOH.


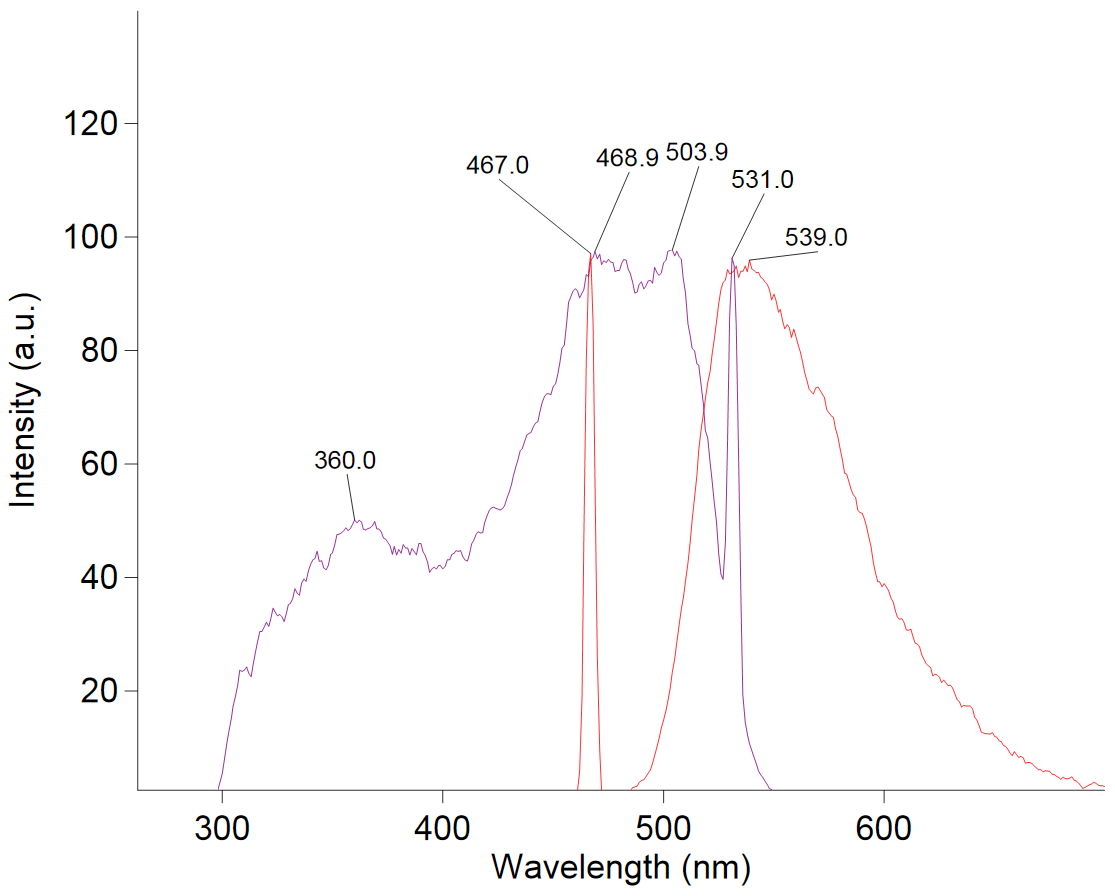


**Figure S16.** Compound (**28**) in absolute EtOH.

**Plots for Quantum Yield Determinations of Singapore Greens**

**Figure S17** Fluorescein standard 2 Absorbance vs Fluorescence intensity

**Figure S18** (15) Absorbance vs Fluorescence intensity Y = 0.647431877

**Figure S19.** (15) Concentration vs Absorbance Ɛ = 46044 M^-1^cm^-1^

**Figure S20**. (16) Absorbance vs Fluorescence intensity QY = 0.561098083

**Figure S21** (16) Concentration vs Absorbance Ɛ = 25695 M^-1^cm^-1^

**Figure S22.** (17) Absorbance vs Fluorescence intensity QY = 0.63144219

**Figure S23** (17) Concentration vs Absorbance Ɛ = 26450 M^-1^cm^-1^

**Figure S24** (18) Absorbance vs Fluorescence intensity QY = 0.475715251

**Figure S25** (18) Concentration vs Absorbance Ɛ = 44527 M^-1^cm^-1^

**Figure S26** Fluorescein standard 3 Absorbance vs Fluorescence intensity

**Figure S27** (19) Absorbance vs Fluorescence intensity QY = 0.014651641

**Figure S28** (19) Absorbance vs Concentration Ɛ = 35411 M^-1^cm^-1^

**Figure S29** (20) Absorbance vs Fluorescence intensity QY = 0.390550111

**Figure S30** (20) Concentration vs Absorbance Ɛ = 22822 M^-1^cm^-1^

**Figure S31** (21) Absorbance vs Fluorescence intensity QY = 0.009824684

**Figure S32** (21) Concentration vs Absorbance Ɛ = 38279 M^-1^cm^-1^

**Figure S33** (22) Absorbance vs Fluorescence intensity QY = 0.564067922

**Figure S34** (22) Concentration vs Absorbance Ɛ = 36186 M^-1^cm^-1^

**Figure S35** (23) Absorbance vs Fluorescence intensity QY = 0.652890671

**Figure S36** (23) Concentration vs Absorbance Ɛ = 17178 M^-1^cm^-1^

**Figure S37** Fluorescein standard 4 Absorbance vs Fluorescence intensity

**Figure S38** (25) Absorbance vs Fluorescence intensity QY = 0.120883283

**Figure 39** (25) Concentration vs Absorbance Ɛ = 225 M^-1^cm^-1^

**
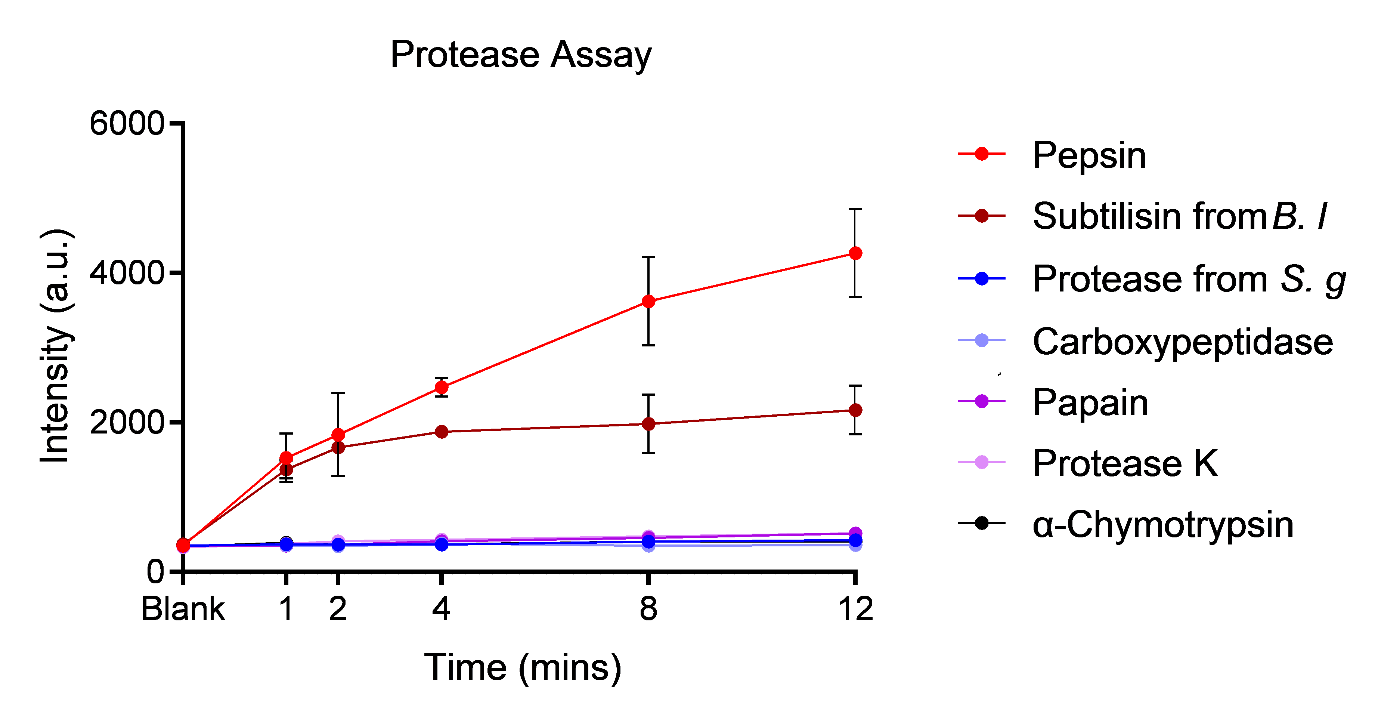
**

**Figure S40.** Increase in fluorescence over time for protease assays against (**25**), error bars represent standard deviation, data is representative of two independent experiments. Blank corresponds to presence of compound (**25**) in each of the respective enzyme activation solvent conditions, in the absence of the enzyme in question. Plotted values for blank are after 24 hours.
